# Heat illness data strengthens vulnerability maps

**DOI:** 10.1186/s12889-021-12097-6

**Published:** 2021-11-03

**Authors:** Jihoon Jung, Christopher K. Uejio, Kristina W. Kintziger, Chris Duclos, Keshia Reid, Melissa Jordan, June T. Spector

**Affiliations:** 1 Department of Environmental and Occupational Health Sciences, University of Washington, Seattle, WA USA; 2 Department of Geography, Florida State University, Tallahassee, FL USA; 3 Department of Public Health, University of Tennessee, Knoxville, TN USA; 4 Public Health Research Unit, Division of Community Health Promotion, Florida Department of Health, Tallahassee, FL USA

**Keywords:** Heat vulnerability, Case-crossover analysis, Spatial lag model, Social determinants of health

## Abstract

**Background:**

Previous extreme heat and human health studies have investigated associations either over time (e.g. case-crossover or time series analysis) or across geographic areas (e.g. spatial models), which may limit the study scope and regional variation. Our study combines a case-crossover design and spatial analysis to identify: 1) the most vulnerable counties to extreme heat; and 2) demographic and socioeconomic variables that are most strongly and consistently related to heat-sensitive health outcomes (cardiovascular disease, dehydration, heat-related illness, acute renal disease, and respiratory disease) across 67 counties in the state of Florida, U. S over 2008–2012.

**Methods:**

We first used a case-crossover design to examine the effects of air temperature on daily counts of health outcomes. We employed a time-stratified design with a 28-day comparison window. Referent periods were extracted from ±7, ±14, or ± 21 days to address seasonality. The results are expressed as odds ratios, or the change in the likelihood of each health outcome for a unit change in heat exposure. We then spatially examined the case-crossover extreme heat and health odds ratios and county level demographic and socioeconomic variables with multiple linear regression or spatial lag models.

**Results:**

Results indicated that southwest Florida has the highest risks of cardiovascular disease, dehydration, acute renal disease, and respiratory disease. Results also suggested demographic and socioeconomic variables were significantly associated with the magnitude of heat-related health risk. The counties with larger populations working in farming, fishing, mining, forestry, construction, and extraction tended to have higher risks of dehydration and acute renal disease, whereas counties with larger populations working in installation, maintenance, and repair workers tended to have lower risks of cardiovascular, dehydration, acute renal disease, and respiratory disease. Finally, our results showed that high income counties consistently have lower health risks of dehydration, heat-related illness, acute renal disease, and respiratory disease.

**Conclusions:**

Our study identified different relationships with demographic/socioeconomic variables for each heat-sensitive health outcome. Results should be incorporated into vulnerability or risk indices for each health outcome.

**Supplementary Information:**

The online version contains supplementary material available at 10.1186/s12889-021-12097-6.

## Introduction

The frequency of heat waves has increased in Europe, Asia, and Australia [1, 2]. Meehl and Tebaldi (2004) and Beniston et al. (2007) also projected that future heat waves will be more intense, more frequent, and longer lasting in Europe and North America [3, 4]. Multiple studies reported that extreme heat elevates the mortality and morbidity risk from cardiovascular [5–7], respiratory [8, 9], heat-related [10, 11], and renal disease [12, 13]. With an increase in the total number of older adults worldwide, the impact of heat waves is predicted to get worse in the future.

Heat stress is not only influenced by heat exposure, but also by demographic factors and social determinants of health (SDOH). Demographic factors, such as age [14–17], sex [18–20], and race/ethnicity [21, 22], influence the adaptive capacity of individuals to extreme heat exposure through physiological processes or socioeconomic status. SDOH also affect the impact of heat waves on human health. Financial assets (e.g. money, vehicles, housing), human capital (e.g. education), and social capital (e.g. churches, neighborhood associations, friend groups, etc.) readily influence people’s well-being and vulnerability [23–25]. For example, employment status (e.g. unemployment rate, labor force) and occupation type (e.g. agriculture and construction) are highly associated with vulnerability to heat waves since it could represent the amount of human and financial capital and the magnitude of heat exposures during hazards [26].

Historically, almost all extreme heat and human health studies have examined associations either over time (e.g. case-crossover or time series designs) or across geographic areas (e.g. spatial analysis). This analytical choice sometimes narrowed research scopes and areas due to limited health outcomes and patient information restricted by health information privacy. In case-crossover designs, each case serves as his or her own control, which strengthens causal inferences [27]. Although the case-control study design implicitly controls for time-invariant individual confounders (e.g. age, sex), it provides limited information about heat vulnerability between geographic areas. In addition, only a very limited number of variables such as age, sex, and race/ethnicity, excluding most socioeconomic variables (e.g. occupation, income, education), were studied due to privacy issues. Instead of focusing on time, geographic studies often map extreme heat vulnerability using a combination of pre-selected SDOH. In general, this technique can identify areas with disproportionately high heat-related mortality and morbidity rates [28, 29]. However, validation studies suggest some of the pre-selected SDOH are not consistently associated with health outcomes [30, 31].

Our study examines the relative importance of SDOH for five different heat-related health outcomes (cardiovascular disease, dehydration, heat-related illness, acute renal disease, and respiratory disease) over 2008–2012. The study sequentially combines a case-crossover design and spatial analysis to identify the counties in Florida most vulnerable to extreme heat and the strongest and most consistent SDOH related to heat-sensitive health outcomes. This methodology attempts to address research gaps by providing more insight into SDOH heat sensitivities and increasing the accuracy of vulnerability mapping.

We first calculate the increase in likelihood of health outcomes when temperature rises through a case-crossover design for 67 counties in the state of Florida. Many case-crossover studies in the past averaged results over an entire state or multi-county region. Then, the study regresses SDOH on the case-crossover odds ratios (ORs) to identify the most consistent and strongest SDOH metrics. This alternative method of identifying place-based risk factors may be more accurate than vulnerability indices created with pre-selected risk factors. This research may increase the accuracy of vulnerability mapping and help identify high risk groups who can further benefit from public health interventions.

## Methods

### Study location and period

The study area included all 67 counties in the state of Florida, U.S. The analysis period only included the warm season (May through September) from 2008 to 2012. Our analytic framework used weather, health, and demographic and socioeconomic variables in Florida. Below, we detail each component of our analytic framework as it was applied to measure SDOH vulnerability at the county level.

### Weather data

We utilized model-derived weather data, North America Land Data Assimilation System phase 2 (NLDAS-2) data. Observational data may be more accurate than model-derived data. However, this data type might not be ideal for studying broad areas, such as state and country, due to limited observational network coverages. For example, there were 92 weather stations for the research period in Florida, which only covered 40 out of 67 counties (Global Surface Summary of the Day; https://gis.ncdc.noaa.gov/maps/ncei/cdo/daily). In addition, weather station data have different systematic errors depending on weather station networks [32].

On the other hand, the NLDAS-2 data provide spatially and temporally consistent model-derived weather data from 1979 to the present. Even though coastal areas tend to have higher biases, up to − 1.48 °C for maximum temperature, no significant biases were found from heat island effects and land cover (water fraction) [33]. The data also produce a wide range of atmospheric variables, including temperature, humidity, pressure, wind, and precipitation, based on a 12-km spatial and hourly temporal resolution. The NLDAS-2 data were originally interpolated from North American Regional Reanalysis data, which had a 32-km spatial resolution and 3-h temporal frequency [34].

We considered daily maximum, mean, and minimum temperature from NLDAS-2 to find the association between heat exposure and daily number of emergency department (ED) visits and hospital admissions. We also employed daily maximum Heat Index (HI) using Eq. 1 with some adjustments [35, 36]. To increase the representativeness of heat exposure in the analysis, we developed population-weighted heat exposure metrics using ZIP code centroids and 2010 census block level population data. More details can be found in Jung et al. [37].

Eq. 1 $$ \mathrm{HI}=-42.379+2.049015\;\mathrm{T}+10.143331\mathrm{R}-0.224755\mathrm{TR}-6.83783\times {10}^{-3}\;{\mathrm{T}}^2-5.481717\times {10}^{-2}{\mathrm{R}}^2+1.22874\times {10}^{-3}{\mathrm{T}}^2\mathrm{R}+8.5282\times {10}^{-4}{\mathrm{T}\mathrm{R}}^2-1.99\times {10}^{-6}{\mathrm{T}}^2{\mathrm{R}}^2 $$

, where T is air temperature (°F) and R is relative humidity (%).

### Health data

The Agency for Health Care Administration (AHCA) is Florida’s principal health policy and planning entity that administers Medicaid, licenses health care facilities, and distributes healthcare data. AHCA provided deidentified ED visit and hospital admission data which contained primary and secondary diagnoses and billing information at the individual level. Based on the International Classification of Diseases Clinical Modification 9th revision, we selected all ED and hospitalization data for cardiovascular disease (390–459), dehydration (276.51), heat-related illness (992, E900.0, E900.1, E900.9), acute renal disease (584.5–584.9), and respiratory disease (460–519). We then removed subsequent visits by the same patient in the following 7 days (dehydration, heat-related illness, respiratory disease) or 28 days (cardiovascular disease and acute renal disease) to increase independence between illnesses with deidentified (masked) SSNs. The Florida Department of Health Human Subjects Committee approved the project (# 2020–033-UW).

### Demographic and socioeconomic data

Publicly available rolling five-year ACS data (2008–2012) and U.S. Decennial Census data (2010) provided demographic and socioeconomic data. The U.S. Census Bureau conducts nationwide surveys with different purposes. U.S. Decennial Census data count the number of people for the purpose of congressional apportionments every 10 years. ACS data are designed to measure the annual changes in socioeconomic characteristics of the U.S. Population [38]. These datasets share similar questions on demographic (e.g. age, sex, race/ethnicity), social, economic, and housing characteristics. The surveys follow different residence rules. Whereas U.S. Decennial Census data follow a “usual residence” rule, ACS data use a “current residence” rule. In addition, the time of year when data are collected varies in the datasets. While ACS data use all 12 months of data, the U.S. Decennial Census data describe the data collected from March through June (when census mail returns are received).

Based on previous papers on SDOH, we selected a total number of 30 demographic and socioeconomic variables which include age, sex, race/ethnicity, employment, wealth, education, housing, single-parents family, and urbanicity (Table 1) [39, 40]. We primarily used ACS data to avoid potential uncertainties and errors from combining two different data sets. Only two variables, percent of population who are 65 and over in nursing facilities and percent of population living in rural block groups, were attained from U.S. Decennial Census data, since ACS did not collect this information. More details on variable selection can be found in supplemental material.

**Table 1:** List of demographic and socioeconomic variables at the county level. All data are downloaded from ACS data except for two variables using U.S. Decennial Census data (marked with *)

	Description	Unit
Age	Median age	Year
	Under 5 years	Percent
	Over 65 years	
	*Over 65 years in nursing facilities	
Sex	Female	Percent
Race/ Ethnicity	Non-Hispanic White	Percent
	Non-Hispanic Black	
	Non-Hispanic other races (American Indian, Asian, Native Hawaiian, or other races)	
	Hispanic	
Employment (16 years and older)	Labor force	Percent
	Unemployment rate	
	Farming, fishing, mining, and forestry	
	Construction and extraction	
	Installation, maintenance, and repair	
	Services	
Wealth (assets, capital)	Average income earned per person (Per capita income)	Dollar
	Median household income	
	Median gross rent	
	Median house value	
	Households earning $10,000 or less	Percent
	Households earning $200,000 or more	
	Households receiving food stamps/SNAP	
	Population 16 years and older below poverty level	
	Housing units with no automobile	
Education	Population 25 years or older with less than a high school diploma	Percent
	Population 5 years and older speaking English as a second language with limited English proficiency (those who speak English not very well or not at all)	
Housing	Median year structure built	Year
	Housing units that are mobile homes	Percent
One-parent	Children under 18 years living in one-parent families	Percent
Urbanicity	*Population living in rural block groups	Percent

### Analytic approach

Our analytic framework can be largely divided into two steps. First, we respectively calculated the county level ORs up to 10 days lag for five heat-related health outcomes (i.e. cardiovascular disease, dehydration, heat-related illness, acute renal disease, and respiratory disease) with daily ED visits and hospital admissions using a case-crossover design. Next, we investigated the relationship between the derived ORs at lag 0 (concurrent day) and social determinants using either multiple linear regression (MLR) or spatial lag models at the county level. Our models used the ORs as a dependent variable and demographic and socioeconomic variables as independent variables.

### Case-crossover analysis

We employed a semi-symmetric bidirectional time stratified case-crossover study design to investigate the impact of extreme heat on daily number of ED visits and hospital admissions. This design is especially useful when studying transient effects (e.g. extreme heat and air pollution) on the risk of acute health events. We compared the temperature metrics when a patient visited the ED or hospital (case) with the temperature metrics when the patient did not seek healthcare (control) within a 28-day comparison window. We extracted controls from ±7, ±14, or ± 21 days to address the weekly and seasonal cycles of health outcomes. Because each case acts as his/her own control, all time-invariant confounders were controlled for by design.

We used conditional logistic regression models to determine ORs for health outcomes per unit change (°C) at the county level. An OR significantly greater (less) than 1 indicates a unit change in temperature increases (decreases) the likelihood of health outcomes. After separately examining four types of heat exposure metrics (maximum, minimum, mean temperature, and maximum HI) and temporal lags (0 to 10 days prior), we selected a concurrent day mean temperature, which exhibited the best fit and lowest AIC with health outcomes (Supplemental Table 1).

### Spatial SDOH analysis

The study examined the relationship between county level case-crossover ORs and demographic/socioeconomic variables (Table 1). We initially checked for potential multicollinearity between the 30 demographic/socioeconomic variables. Strong and consistent associations between two or more independent variables may undermine the statistical significance of an independent variable and give inflated or wrong coefficients. To avoid multicollinearity, we deleted twelve variables with bivariate Pearson correlation coefficients > 0.7: median age, non-Hispanic White, median household income, median gross rent, median house value, households earning $200,000 or more, households receiving food stamps/SNAP, population 16 years and older below poverty level, population 25 years or older with less than a high school diploma, population five years of age and older speaking English as a second language with limited English proficiency, housing units that are mobile homes, and population living in rural block groups [41]. All Pearson correlation coefficients between independent variables can be found in Supplemental Table 2.

With the remaining variables, we separately built ten different statistical models for the five different health outcomes of ED visits and hospital admissions. The dependent variable was the ORs derived from case-crossover results, and independent variables were the remaining 18 demographic/socioeconomic variables. Each model used a different set of independent variables identified through a backward stepwise AIC variable selection procedure. This selection process begins with a full model which contains all 18 independent variables. Then, the model sequentially removes any independent variables which do not improve model fit based on AIC. This removal process stops when there is no further model improvement, and the final model tends to contain the lowest AIC value.

We considered both aspatial and spatial models to control for residual spatial autocorrelation. Spatial autocorrelation refers to geographically nearby values tending to have similar values. Spatial autocorrelation can introduce biases or errors in a study of this type [42, 43]. We first started with aspatial MLR models to relate the ORs for each health outcome and demographic/socioeconomic variables. We then checked for violations of model assumptions, specifically in normality of residuals using the Kolmogorov-Smirnov normality test and for spatial autocorrelation using the Global Moran’s I. Global Moran’s I is a commonly used term to measure the degree of clustering. In general, a value near 1, 0, and − 1 indicate clustering, random, and dispersion, respectively. If spatial autocorrelation was found in the model residuals or if spatial models had better AICs than aspatial model, we employed spatial lag models. Otherwise, we used aspatial MLR models.

For spatial lag models, we used a basic first-order queen contiguity-based approach to define neighborhood weights without row standardization. Spatial lag models can estimate the impact of local (direct impact), spillover (indirect impact, neighbor), and total impact. We only reported global average total impacts and *p*-values since the purpose of the model was controlling for residual autocorrelation as opposed to providing insight into spatial processes. All calculations were done with the R (V. 3.6.2) statistical analysis and computing program. Spdem and spatialreg packages were used to create spatial lag models and to calculate Moran’s I Monte Carlo estimated *p*-values. Because of the limited number of cases, we were not able to make statistical models for heat-related illness hospitalization and acute renal disease ED visits for the second step.

## Results

### Descriptive summaries

Table 2 displays the total number of cases and a summary of daily mean temperature for cases and controls at lag 0. Overall, cardiovascular disease and respiratory disease had the highest number of cases, followed by dehydration, acute renal disease, and heat-related illness. The number of hospitalizations was higher than ED cases for cardiovascular illness, dehydration, and acute renal disease, while the opposite was observed for heat-related illness and respiratory disease. Cases tended to have slightly higher average and minimum values of daily mean temperature than controls throughout five illnesses. In particular, heat-related illness showed the greatest difference of 0.6 °C in average values of daily meant temperature for both ED visits and hospitalizations. In addition, we observed a relatively large difference from the minimum value of daily mean temperature for heat-related illness (ED: 6.0 °C; hospitalization: 2.7 °C). Otherwise, there were no large differences in maximum values of daily mean temperature.

**Table 2:** Total number of cases and a summary of mean temperature for cases and controls at lag 0. ED and HSP respectively stand for emergency department visits and hospital admissions

			Total cases	Minimum (°C)	Average (°C)	Maximum (°C)
Cardiovascular disease	ED	Case	1,908,926	12.4	27.6	33.7
		Control	5,726,778	12.1	27.4	33.7
	HSP	Case	2,330,824	12.4	27.6	33.7
		Control	6,992,472	12.1	27.4	33.7
Dehydration	ED	Case	120,432	13.1	27.6	33.7
		Control	361,296	12.2	27.3	33.7
	HSP	Case	251,649	12.4	27.6	33.7
		Control	754,947	12.1	27.4	33.7
Heat-related illness	ED	Case	13,708	18.6	28.4	33.7
		Control	41,124	12.6	27.8	33.7
	HSP	Case	2849	17.8	28.5	33.5
		Control	8547	15.1	27.9	33.5
Acute renal disease	ED	Case	10,135	14.0	27.7	33.5
		Control	30,405	12.2	27.4	33.7
	HSP	Case	337,879	12.4	27.6	33.7
		Control	1,013,637	12.1	27.4	33.7
Respiratory disease	ED	Case	1,528,839	12.4	27.5	33.7
		Control	4,586,517	12.1	27.3	33.7
	HSP	Case	1,206,701	12.4	27.6	33.7
		Control	3,620,103	12.1	27.4	33.7

Table 3 summarizes the average heat vulnerability factors at the county level. Note that the average of risk factors at the county level is not equivalent to the average for the entire state. Median age ranged from 29.6 to 63.0 with an average of 42.0 years. Vulnerable people aged under 5 and over 65, respectively, was 5.6 and 18.3% of the total county population. The female population (48.7%) was slightly smaller than male population. Non-Hispanic White (79.3%) comprised the highest portion of the population, followed by non-Hispanic Black (14.5%), and non-Hispanic other races (6.2%). Even though the county level average Hispanic population was 12.5%, some counties such as Miami-Dade (64.6%), Hendry (49.0%), and Osceola County (45.8%) showed a higher proportion of Hispanic people (Supplemental Table 3). On average, at least 10% of the people in a county worked outside, including workers in agriculture, fishing, mining, and forestry (2.5%) and construction and extraction (7.8%). Median household income was $44,269, with 8.2 and 2.5% of households earning less than $10,000 and more than $200,000 respectively. Approximately 10 % of people used food stamps, and 16.0% of people were below the federal poverty level. More detailed information by county can be found in Supplemental Table 3.

**Table 3:** County summary statistics of demographic and socioeconomic variables

Variable	Unit	Mean	SD	Max	Min	Range
Median age	Year	42.0	5.9	63.0	29.6	33.4
Under 5 years	Percent	5.6	1.0	8.0	2.4	5.6
Over 65 years		18.3	6.8	44.5	9.5	35.0
Over 65 years in nursing facilities		0.4	0.2	1.2	0.0	1.2
Female		48.7	3.7	52.5	35.6	16.8
Non-Hispanic White		79.3	10.1	93.4	37.0	56.3
Non-Hispanic Black		14.5	9.5	55.5	2.9	52.6
Non-Hispanic other races (American Indian, Asian, Native Hawaiian, other races)		6.2	3.1	20.1	2.0	18.0
Hispanic		12.5	12.0	64.6	2.1	62.5
Labor force		53.7	8.3	68.8	26.2	42.6
Unemployment rate		12.6	2.5	20.6	7.5	13.1
Farming, fishing, mining, and forestry		2.5	4.5	23.2	0.0	23.1
Construction and extraction		7.8	2.2	14.7	2.9	11.7
Installation, maintenance, and repair		4.1	1.1	8.3	1.7	6.6
Services		20.5	3.4	35.5	14.4	21.1
Average income earned per person (Per capita income)	1000 Dollar	23.1	5.9	37.0	13.7	23.4
Median household income		44.3	7.5	62.7	31.7	31.0
Median gross rent		0.8	0.2	1.3	0.5	0.7
Median house value		169.8	70.4	503.9	75.7	428.2
Households earning $10,000 or less	Percent	8.2	2.8	15.8	4.1	11.7
Households earning $200,000 or more		2.5	1.7	8.1	0.1	8.0
Households receiving food stamps/SNAP		9.7	4.0	18.5	3.8	14.7
Population 16 years and older below poverty level		16.0	4.9	27.6	8.5	19.1
Housing units with no automobile		6.3	1.9	11.2	3.0	8.2
Education (less than a high school diploma)		17.8	7.0	40.1	7.2	32.9
English proficiency		3.6	4.4	22.3	0.2	22.1
Median year structure built	Year	1986.1	4.7	2000.0	1975.0	25.0
Housing units that are mobile homes	Percent	22.8	14.9	57.2	1.5	55.7
Children under 18 years living in one-parent families		34.3	6.0	58.3	18.6	39.7
Population living in rural block groups		38.6	33.9	100.0	0.0	100.0

### Spatial distribution of ORs

**Fig. 1:**
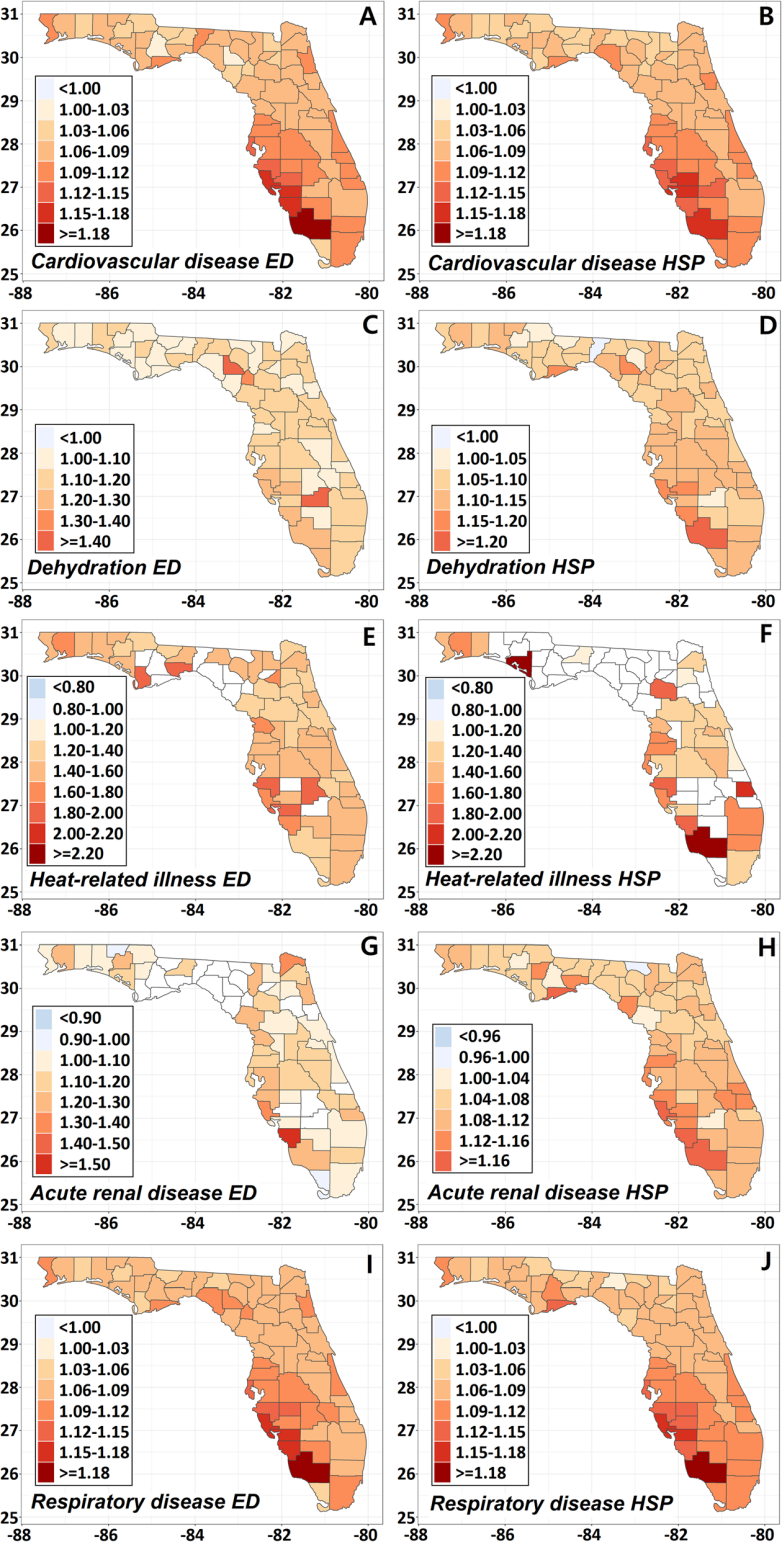
County level ORs at lag 0. Counties having less than 30 cases were colored in white. ED and HSP, respectively, stand for emergency department visits and hospital admissions

Figure 1 shows the spatial patterns of county level ORs at lag 0. Southwest Florida clearly showed higher ORs compared to the rest of Florida for cardiovascular and respiratory illness (Fig. 1 A, B, I, J). ORs for dehydration hospitalization and acute renal disease hospitalization were also higher in southwest Florida (Fig. 1 D, H) and the Florida Panhandle and southwest Florida had higher ORs for heat-related illness (Fig. 1 E, F). However, we were not able to observe any patterns from dehydration ED and acute renal disease ED (Fig. 1 C, G). Sensitivity analyses up to 7 lag days showed similar spatial patterns (Supplemental Fig. 1). All diseases were most sensitive to the concurrent day’s temperature compared to all other seven lag days.

### The relationship between concurrent ORs and SDOH

To find the relationship between the concurrent ORs and SDOH, we tested both aspatial (MLRs) and spatial models (spatial lag model). Based on residual spatial autocorrelation and AIC, we selected spatial lag models for cardiovascular disease ED and respiratory disease ED/hospitalization, and MLRs for cardiovascular disease hospitalization, dehydration ED/hospitalization, heat-related illness ED, Acute renal disease hospitalization. Because of the limited number of cases, we were not able to make statistical models for heat-related illness hospitalization and acute renal disease ED visits. Each model had its own unique set of independent variables representing the lowest AIC.

Table 4 summarizes the regression coefficients and *p*-values corresponding to each demographic/socioeconomic variable and each health outcome. The relationships were generally consistent across the analysis. Counties having more vulnerable demographics including those over 65 and over 65 years in nursing facilities showed higher risks of illnesses. These demographic variables were significantly related with cardiovascular disease (over 65 ED 0.001; over 65 hospitalization: 0.001; over 65 in nursing facilities ED: 0.008). This suggests that 1 % increase in those over 65 and those over 65 in nursing facilities raised a cardiovascular disease ED and hospitalization ORs by 0.001 and ED OR by 0.008 respectively. Those over 65 and over 65 years in nursing facilities were also positively associated with heat-related illness (ED: 0.008), acute renal disease (hospitalization: 0.020), and respiratory disease (ED: 0.012), indicating increases in heat-related illness ED OR, acute renal disease hospitalization OR, and respiratory disease ED OR by 0.008, 0.020, and 0.012 respective with 1 % increase of these populations. The percent of female population showed mixed results, having a positive relationship with dehydration (ED: 0.004, hospitalization: 0.003) and a negative relationship with cardiovascular disease (hospitalization: − 0.001) and heat-related illness (ED: − 0.010).

**Table 4:** Multiple linear regression/spatial lag model results. MLR, ED, and HSP, respectively, stand for multiple linear regression, emergency department visits and hospital admissions. (* ≤ 0.05)

	Cardiovascular disease		Dehydration		Heat-related illness		Acute renal disease		Respiratory disease	
	ED	HSP	ED	HSP	ED	HSP	ED	HSP	ED	HSP
Model type	Spatial lag	MLR	MLR	MLR	MLR	–	–	MLR	Spatial lag	Spatial lag
Under 5 years	0.003*	0.002	–	–	–	–	–	–	–	–
Over 65 years	0.001*	0.001*	–	–	0.008*	–	–	–	–	0.000
Over 65 years in nursing facilities	0.008*	–	–	–	–	–	–	0.020*	0.012*	–
Female	–	−0.001*	0.004*	0.003*	−0.010*	–	–	0.001	–	–
Non-Hispanic Black	–	–	–	–	–	–	–	–	–	0.000*
Non-Hispanic other races (American Indian, Asian, Native Hawaiian, other races)	–	–	–	–	–	–	–	–	–	–
Hispanic	–	–	–	–	–	–	–	–	–	–
Labor force	–	–	–	–	0.007*	–	–	–	–	–
Unemployment rate	−0.001	− 0.001*	–	–	− 0.006	–	–	− 0.002*	–	–
Farming, fishing, mining, and forestry	–	–	0.003*	− 0.001	–	–	–	–	–	–
Construction and extraction	–	–	–	0.005*	–	–	–	0.002*	–	–
Installation, maintenance, and repair	−0.003*	− 0.002*	0.005	−0.009*	–	–	–	− 0.005*	−0.002	− 0.004*
Services	–	− 0.001	0.004*	–	−0.005	–	–	–	–	− 0.001*
Average income earned per person (Per capita income)	–	–	–	−0.001*	− 0.006*	–	–	− 0.001*	–	− 0.001*
Households earning $10,000 or less	–	–	–	–	–	–	–	−0.002*	–	–
Housing units with no automobile	−0.001*	–	–	0.003	–	–	–	–	−0.001*	–
Median year structure built	–	0.000	−0.001	–	–	–	–	–	–	–
Children under 18 years living in one-parent families	0.001*	–	−0.002*	−0.002*	–	–	–	–	–	–

Outdoor workers tended to show positive relationships with heat-related health outcomes, while indoor workers exhibited negative relationships. For example, the percent of people engaged in farming, fishing, mining, and forestry was positively related with dehydration (ED: 0.003). The percent of population employed in construction and extraction was also positively associated with dehydration (hospitalization: 0.005) and acute renal disease (hospitalization: 0.002). This exhibits that 1 % increase in those employed in farming, fishing, mining, and forestry and those employed in construction and extraction, respectively, raised a dehydration ED OR, dehydration hospitalization OR, and acute renal disease hospitalization OR by 0.003, 0.005, and 0.002. On the other hand, the percent of population working in installation, maintenance, and repair tended to show negative relationships with cardiovascular disease (ED: − 0.003, hospitalization: − 0.002), dehydration (hospitalization: − 0.009), acute renal disease (hospitalization: − 0.005), and respiratory disease (hospitalization: − 0.004), suggesting decreased ORs in cardiovascular disease ED by − 0.003, cardiovascular disease hospitalization by − 0.002, dehydration hospitalization by − 0.009, acute renal disease hospitalization by − 0.005, and respiratory disease hospitalization by − 0.004 with 1 % increase of these populations. Somewhat unexpectedly, we found a negative relationship between unemployment rates and cardiovascular disease (hospitalization: − 0.001) and acute renal disease (hospitalization: − 0.002).

As for income, the higher average income per person counties had lower risks of health outcomes (dehydration hospitalization: − 0.001, heat-related illness ED: − 0.006, acute renal disease hospitalization: − 0.001, respiratory disease hospitalization: − 0.001). In other words, one unit increase ($1000) in average income per person decreased dehydration hospitalization OR by − 0.001, heat-related illness ED OR by − 0.006, acute renal disease hospitalization OR by − 0.001, and respiratory disease hospitalization OR by − 0.001. We also observed negative associations between the percent of housing units with no automobile and cardiovascular disease (ED: − 0.001) and respiratory disease (ED: − 0.001). In addition, the percent of one-parent families was positively associated with cardiovascular disease (ED: 0.001) and negatively associated with dehydration (ED: − 0.002, hospitalization: − 0.002). For comparisons of relative importance of independent variables across health outcomes, we created a table showing the full model coefficients and *p*-values, which covers all 18 independent variables in Table 4 (Supplemental Table 4).

## Discussion

This study illustrates the benefits of spatially analyzing heat health sensitivities derived from a case-crossover study design. Our identification of the strongest and most consistent heat risk factors produced different results than “all hazards” vulnerability mapping using pre-selected risk factors. In our study, southwest Florida displays the highest risks of cardiovascular disease, dehydration, acute renal disease, and respiratory disease. The higher proportion of adults over the age of 65, 24.7% according to the U.S. Decennial Census (2010), was notably higher than other regions (13.7 to 19.2%) and may be partially responsible for increased risk. This spatial pattern is notably different from Emrich et al. (2014) which suggested southeast Florida was the most vulnerable areas to natural hazards [44]. Our study used empirical morbidity data to calculate the health risks, whereas Emrich et al. (2014)‘s vulnerability index is based on pre-selected risk factors and not health outcomes [44]. Our results suggest that a priori vulnerability assessments can be improved by more explicitly considering climate sensitive health outcomes. Social vulnerability assessments frequently give equal weight to individual or synthetic (e.g. principal component) risk factors. In contrast, the regression beta coefficients derived from the present study could be used as heat risk factor weights.

In this study, we identified evidence to suggest that demographic and socioeconomic variables influence the magnitude of heat-related health risk. The percent of adults over age 65 in nursing homes was one of the most influential factors for cardiovascular disease, acute renal disease, and respiratory disease. This result is consistent with previous papers [45, 46]. These papers indicate that institutionalized older adults are at higher risk than those not living in institutions during heat waves. For example, Stafoggia et al. (2006) found a higher OR from those living in nursing homes (OR: 1.61, 95% CI: 1.41–1.84) than any other age groups over 65 (65–74: 1.25, 1.12–1.38; 75–84: 1.36, 1.28–1.44; 85–94: 1.49, 1.37–1.63; 95+: 1.58, 1.34–1.85) [45].

Our study also clearly showed differential heat-related health impacts depend on occupation. When temperature increases, the counties having more people working in farming, fishing, mining, forestry, construction, and extraction tended to have higher risks of dehydration and acute renal disease. This is consistent with other research. Moyce (2016) reported that agricultural workers are at high risk of acute kidney disease related to work in hot conditions (adjusted OR 1.34, 95% CI: 1.04–1.74) [47]. High rates of chronic kidney disease were also found from sugarcane workers in Central America, and repeated exposure to heat stress has been implicated as a potential cause [48]. Moreover, temperature raises the risk of dehydration for farm workers [49]. In contrast, the counties having larger populations working in installation, maintenance, and repair tended to have lower risks of cardiovascular, dehydration, acute renal, and respiratory disease. According to the U.S. Bureau of Labor Statistics, those employed in installation, maintenance, and repair occupations (43.0 h/week) had relatively shorter work hours than mining (49.2) and construction (42.6) in 2019 [50]. The Central Statistics Office also reported that workers in the agriculture, forestry, and fishing industries worked 50.4 h/week compared with the national average of 35.7 h/week in 2015 [51]. Those employed in farming, fishing, mining, forestry, construction, and extraction spend a large proportion of time doing heavy physical work outside (e.g. harvest, roofing), which can substantially increase heat exposure. Furthermore, these jobs are more physically demanding than other occupations, and workers may not have control over their work conditions to allow for adequate breaks and cooling.

Our result exhibited that high income counties consistently have lower health risk of dehydration, heat-related illness, acute renal disease, and respiratory disease. Hondula and Barnett (2014) also showed that a 1% increase in the proportion of high-income residents (weekly income > $1600) was associated with 8.5 fewer heat-related hospitalizations in Brisbane, Australia [52]. Other papers support the negative relationships between income and heat-related health risks [53–55]. The relation may exist because people with low incomes are more likely to reside in poor indoor environments with limited cooling [56], have limited access to healthy food [57, 58], and not have health insurance [59, 60].

In addition, we consistently observed negative associations between health outcomes and unemployment rate (cardiovascular disease and acute renal disease) and the percent of housing units with no automobile (cardiovascular disease and respiratory disease). We suspect these factors may impact the number of health care visits through limited health insurance coverage or limited accessibility to health care facilities. In the U.S., where health insurance is frequently connected to employment, healthcare usage is negatively associated with unemployment rate [61–63]. Comber et al. (2011) also presents that car ownership is highly associated with assesses to health care facilities, particularly in rural areas [64]. Furthermore, we found the median building age may not be a significant extreme heat risk factor in Florida for most heat-related health outcomes. Kovach et al. (2015) and Boroushaki (2017) also support that building year is not a main factor after adjusting for population density, age, poverty, and tree canopy [65, 66].

Finally, our novel methodology showed a couple of advantages over traditional methods. First, the approach expands upon current research topics by providing a way to connect heat-health associations with a wide range of neighborhood demographic or socioeconomic variables collected through various surveys such as American Community Survey (ACS). Traditional stratified analysis or models with interaction terms often only examine a limited number of sociodemographic characteristics, such as age, sex, race/ethnicity, due to limited individual sociodemographic data availability. In addition, our study provides the relative importance of each sociodemographic data for different health outcomes. Previous case-crossover analyses typically considered the interaction between heat and a composite vulnerability index comprised of multiple sociodemographic variables. The vulnerability index provides less guidance to public health departments for interventions due to less information on specific sociodemographic risk factors. Furthermore, our approach can explicitly examine the local and neighboring heat impact effect modification by SDOH, which could benefit public health departments in understanding the spatial processes of heat impacts. Traditional case-crossover study designs, which control residual autocorrelation using conditional Poisson regression, would provide limited information on spatial processes.

Our study design suffers from the same limitations as other spatial ecologic studies. First, our study’s county level correlations do not necessarily translate to individual-level associations. For example, even though we found a strong correlation between those over age 65 in nursing homes and cardiovascular disease, this does not mean every individual over 65 in nursing homes has a higher heat cardiovascular risk. Second, the results may be contingent on the county analysis level. The modifiable areal unit problem is commonly generated when point data are aggregated into areal units. We would anticipate somewhat different associations if the same series of analyses were repeated at regional or ZIP-code analysis levels. Finally, our demographic/socioeconomic variables were from two different data sources. Even though most of our data were from ACS, we still included two variables from U.S. Decennial Census data. Since there are differences regarding data collection methods, some uncertainties and errors could be introduced in the analysis.

Our study has several key policy implications for public health practitioners and policy makers. First, our results show that each health outcome has a different relationship with various demographic and socioeconomic variables. Current warning systems and occupational health standards for heat waves (e.g. California, Washington, Oregon State rules for heat waves) are mostly based on a one-size-fits-all criteria (e.g. 100 °F threshold). This study provides strong evidence that we may need to devise an individual vulnerability or risk index for each health outcome. Second, we provide the relative importance of demographic and socioeconomic variables for each health outcome. This information provides important background for determining the weights of each variable when making vulnerability maps or indexes. Given that previous maps and indexes typically assumed equal weights without an objective way to assign a weight to each variable, our results may improve current vulnerability products. In addition, these results could prioritize prevention efforts, prepare occupational health and safety guidelines, and plan health care resource (e.g., emergency department and hospital access and capacity planning [67]). Third, our method allows investigators to expand the scope of their research, and test other possible mechanisms connecting health outcomes and sociodemographic variables. Most previous studies only investigated basic demographic variables such as age, sex, race/ethnicity, due to limited individual sociodemographic information contained in electronic medical records. With our method, demographic and socioeconomic data at various regional levels (e.g. county, ZIP code) can be connected with health outcomes. Finally, our method provides more in-depth information about the spatial processes of each demographic and socioeconomic variable. Since each variable has a different level of direct or indirect impacts with neighbors, different strategies are needed for each variable. Our results will improve health equity and reduce the overall public health burden of negative health effects from heat exposures.

## Supplementary Information

**Additional file 1:** Supplemental materials. Tables S1-S4, Figure S1.

## Data Availability

Weather data are available from https://disc.gsfc.nasa.gov/datasets?keywords=NLDAS. Demographic and socioeconomic variables are available from https://www.census.gov. the Florida health data can be accessed for valid reasons by qualified health researchers. The Agency for Health Care Administration data website is https://www.floridahealthfinder.gov/researchers/orderdata/order-data.aspx and the email address is contactus@ahca.myflorida.com.

## Abbreviations

AHCA: Agency for Health Care Administration
ACS: American Community Survey
ED: Emergency department
HI: Heat Index
MLR: Multiple linear regression;
NLDAS-2: North America Land Data Assimilation System phase 2
OR: Odds ratio
SDOH: Social determinants of health
