# Supplementary material for: Heat illness data strengthens vulnerability maps
Source: BMC Public Health. 2021 Nov 3;21:1999. doi: 10.1186/s12889-021-12097-6 (PMC8567677; doi:10.1186/s12889-021-12097-6)
Supplement: Supplementary file 1 — Additional file 1: Supplemental materials. Tables S1-S4, Figure S1. [file 12889_2021_12097_MOESM1_ESM.docx]

**Supplemental materials**

**Model variable selection justifications**

Demographic factors influence the adaptive capacity of individuals to extreme heat exposure. Multiple papers reported children under 5 years and the elderly over 65 years are at greatest risk during heat waves (Knowlton et al., 2009; Turner et al., 2013; Wang et al., 2012). Hajat et al. (2007) found a much higher risk for nursing home elderly patients than those living at home (Hajat et al., 2007). For sex, van Steen et al. (2019) indicated that older adult women are at higher risk than men (van Steen et al., 2019). The majority of European studies have also supported that females are at high risk for physiological and social reasons (Kovats and Hajat, 2008). However, for some working age groups, males tend to show higher risks, possibly because they are more likely to work outside (Bai et al., 2014). For race/ethnicity, previous studies described that African Americans have higher risks of death during heat waves, which may be more related to socioeconomic disparities or housing conditions (Kaiser et al., 2007; O’Neill et al., 2005).

Socioeconomic factors also affect the magnitude of heat waves on human health. Financial assets (e.g. money, cars, houses), human capital (e.g. education), and social capital (e.g. churches, neighborhood associations, friend groups, etc.) readily influence people’s well-being and vulnerability (Kim et al., 2011; Medina-Ramón et al., 2006; O’Neill et al., 2003). These resources could boost adaptive capacity before/during/after heat waves. For example, Son et al. (2011) found a higher heat-related death risk for those with lower or no education (Son et al., 2011). Housing characteristics, including median year structure built and mobile homes housing unit, appear to adversely impact human health (Sakka et al., 2012). Employment status (e.g. unemployment rate, labor force) is also highly associated with vulnerability to heat waves since it could represent the amount of human and financial capital during hazards (Anderson and Bell, 2009). Multiple studies support that heat waves adversely affect low-income people (Berry et al., 2010; Harlan et al., 2006; Sakka et al., 2012). Occupation type additionally influences the magnitude of heat risk and exposure. Outdoor workers such as construction, extraction, and agriculture workers show higher risks than other occupations (Fleischer et al., 2013; Petitti et al., 2013).

Anderson, B.G., Bell, M.L., 2009. Weather-related mortality: how heat, cold, and heat waves affect mortality in the United States. Epidemiology 20, 205.

Bai, L., Ding, G., Gu, S., Bi, P., Su, B., Qin, D., Xu, G., Liu, Q., 2014. The effects of summer temperature and heat waves on heat-related illness in a coastal city of China, 2011-2013. Environ. Res. 132, 212–219. https://doi.org/10.1016/j.envres.2014.04.002

Berry, H.L., Bowen, K., Kjellstrom, T., 2010. Climate change and mental health: A causal pathways framework. Int. J. Public Health 55, 123–132. https://doi.org/10.1007/s00038-009-0112-0

Fleischer, N.L., Tiesman, H.M., Sumitani, J., Mize, T., Amarnath, K.K., Bayakly, A.R., Murphy, M.W., 2013. Public health impact of heat-related illness among migrant farmworkers. Am. J. Prev. Med. 44, 199–206.

Hajat, S., Kovats, R.S., Lachowycz, K., 2007. Heat-related and cold-related deaths in England and Wales: who is at risk? Occup. Environ. Med. 64, 93–100.

Harlan, S.L., Brazel, A.J., Prashad, L., Stefanov, W.L., Larsen, L., 2006. Neighborhood microclimates and vulnerability to heat stress. Soc. Sci. Med. 63, 2847–2863.

Kaiser, R., Le Tertre, A., Schwartz, J., Gotway, C.A., Daley, W.R., Rubin, C.H., 2007. The effect of the 1995 heat wave in Chicago on all-cause and cause-specific mortality. Am. J. Public Health 97, S158–S162.

Kim, G., Worley, C.B., Allen, R.S., Vinson, L., Crowther, M.R., Parmelee, P., Chiriboga, D.A., 2011. Vulnerability of older Latino and Asian immigrants with limited English proficiency. J. Am. Geriatr. Soc. 59, 1246–1252.

Knowlton, K., Rotkin-Ellman, M., King, G., Margolis, H.G., Smith, D., Solomon, G., Trent, R., English, P., 2009. The 2006 California heat wave: impacts on hospitalizations and emergency department visits. Environ. Health Perspect. 117, 61–67.

Kovats, R.S., Hajat, S., 2008. Heat Stress and Public Health: A Critical Review. Annu. Rev. Public Health 29, 41–55. https://doi.org/10.1146/annurev.publhealth.29.020907.090843

Medina-Ramón, M., Zanobetti, A., Cavanagh, D.P., Schwartz, J., 2006. Extreme temperatures and mortality: assessing effect modification by personal characteristics and specific cause of death in a multi-city case-only analysis. Environ. Health Perspect. 114, 1331–1336.

O’Neill, M.S., Zanobetti, A., Schwartz, J., 2005. Disparities by race in heat-related mortality in four US cities: The role of air conditioning prevalence. J. Urban Heal. 82, 191–197. https://doi.org/10.1093/jurban/jti043

O’Neill, M.S., Zanobetti, A., Schwartz, J., 2003. Modifiers of the temperature and mortality association in seven US cities. Am. J. Epidemiol. 157, 1074–1082.

Petitti, D.B., Harlan, S.L., Chowell-Puente, G., Ruddell, D., 2013. Occupation and environmental heat-associated deaths in Maricopa County, Arizona: A case-control study. PLoS One 8, e62596.

Sakka, A., Santamouris, M., Livada, I., Nicol, F., Wilson, M., 2012. On the thermal performance of low income housing during heat waves. Energy Build. 49, 69–77.

Son, J.-Y., Lee, J.-T., Anderson, G.B., Bell, M.L., 2011. Vulnerability to temperature-related mortality in Seoul, Korea. Environ. Res. Lett. 6, 34027.

Turner, L.R., Connell, D., Tong, S., 2013. The effect of heat waves on ambulance attendances in Brisbane, Australia. Prehosp. Disaster Med. 28, 482.

van Steen, Y., Ntarladima, A.-M., Grobbee, R., Karssenberg, D., Vaartjes, I., 2019. Sex differences in mortality after heat waves: are elderly women at higher risk? Int. Arch. Occup. Environ. Health 92, 37–48.

Wang, X.Y., Barnett, A.G., Yu, W., FitzGerald, G., Tippett, V., Aitken, P., Neville, G., McRae, D., Verrall, K., Tong, S., 2012. The impact of heatwaves on mortality and emergency hospital admissions from non-external causes in Brisbane, Australia. Occup. Environ. Med. 69, 163–169.

**Supplemental Table 1.** AICs for four different types of heat exposures. ED and HSP respectively stand for emergency department visits and hospital admissions.

|  | Lag | Cardiovascular disease | | Dehydration | | Heat-related illness | | Acute renal disease | | Respiratory disease | |
| --- | --- | --- | --- | --- | --- | --- | --- | --- | --- | --- | --- |
|  |  | ED | HSP | ED | HSP | ED | HSP | ED | HSP | ED | HSP |
| Maximum | 0 | 5,451,235 | 6,687,019 | 369,545 | 732,107 | 39,965 | 8,171 | 29,144 | 954,726 | 5,040,328 | 3,529,228 |
|  | 1 | 5,452,231 | 6,686,589 | 369,895 | 732,318 | 40,902 | 8,246 | 29,165 | 954,773 | 5,041,072 | 3,529,126 |
|  | 2 | 5,452,480 | 6,687,489 | 370,130 | 732,604 | 41,639 | 8,408 | 29,203 | 954,982 | 5,041,598 | 3,529,609 |
|  | 3 | 5,451,848 | 6,687,515 | 370,283 | 732,819 | 41,894 | 8,518 | 29,234 | 955,166 | 5,041,120 | 3,529,557 |
|  | 4 | 5,451,636 | 6,687,898 | 370,359 | 733,014 | 42,035 | 8,551 | 29,231 | 955,296 | 5,041,443 | 3,529,798 |
|  | 5 | 5,451,931 | 6,688,397 | 370,495 | 733,167 | 42,115 | 8,576 | 29,231 | 955,518 | 5,042,117 | 3,529,944 |
|  | 6 | 5,452,159 | 6,688,125 | 370,663 | 733,165 | 42,156 | 8,586 | 29,254 | 955,561 | 5,042,345 | 3,529,745 |
|  | 7 | 5,453,575 | 6,689,777 | 370,753 | 733,355 | 42,160 | 8,589 | 29,264 | 955,857 | 5,043,704 | 3,530,543 |
|  | 8 | 5,454,032 | 6,690,686 | 370,760 | 733,395 | 42,151 | 8,589 | 29,266 | 955,984 | 5,044,043 | 3,531,015 |
|  | 9 | 5,454,441 | 6,691,310 | 370,775 | 733,354 | 42,139 | 8,588 | 29,258 | 956,070 | 5,044,414 | 3,531,403 |
|  | 10 | 5,455,614 | 6,692,628 | 370,804 | 733,543 | 42,135 | 8,587 | 29,257 | 956,129 | 5,045,317 | 3,532,084 |
| Average | | 5,452,835 | 6,688,858 | 370,405 | 732,986 | 41,754 | 8,492 | 29,228 | 955,460 | 5,042,500 | 3,530,187 |
| Minimum | 0 | 5,444,065 | 6,679,799 | 369,228 | 731,663 | 40,922 | 8,401 | 29,121 | 953,762 | 5,033,519 | 3,525,482 |
|  | 1 | 5,444,870 | 6,679,804 | 369,566 | 731,765 | 41,530 | 8,468 | 29,165 | 953,818 | 5,033,879 | 3,525,584 |
|  | 2 | 5,446,692 | 6,682,015 | 370,056 | 732,191 | 41,864 | 8,526 | 29,230 | 954,310 | 5,036,011 | 3,526,586 |
|  | 3 | 5,447,696 | 6,684,031 | 370,176 | 732,567 | 42,000 | 8,544 | 29,229 | 954,757 | 5,036,932 | 3,527,567 |
|  | 4 | 5,449,113 | 6,685,523 | 370,348 | 732,691 | 42,091 | 8,565 | 29,233 | 955,055 | 5,038,714 | 3,528,376 |
|  | 5 | 5,450,532 | 6,686,533 | 370,513 | 732,876 | 42,132 | 8,578 | 29,236 | 955,348 | 5,040,646 | 3,529,018 |
|  | 6 | 5,452,153 | 6,688,396 | 370,647 | 733,159 | 42,138 | 8,588 | 29,250 | 955,631 | 5,043,051 | 3,529,984 |
|  | 7 | 5,453,577 | 6,689,635 | 370,777 | 733,286 | 42,134 | 8,588 | 29,270 | 955,831 | 5,044,667 | 3,530,422 |
|  | 8 | 5,454,146 | 6,689,981 | 370,754 | 733,311 | 42,136 | 8,589 | 29,273 | 955,967 | 5,045,020 | 3,530,737 |
|  | 9 | 5,454,971 | 6,692,043 | 370,782 | 733,490 | 42,123 | 8,589 | 29,265 | 956,161 | 5,045,800 | 3,531,856 |
|  | 10 | 5,455,600 | 6,692,473 | 370,821 | 733,581 | 42,138 | 8,589 | 29,267 | 956,219 | 5,046,346 | 3,532,142 |
| Average | | 5,450,311 | 6,686,385 | 370,334 | 732,780 | 41,928 | 8,548 | 29,231 | 955,169 | 5,040,417 | 3,528,887 |
| Mean | 0 | 5,441,507 | 6,675,576 | 368,537 | 730,664 | 39,648 | 8,161 | 29,066 | 953,032 | 5,030,730 | 3,523,135 |
|  | 1 | 5,443,114 | 6,675,756 | 369,049 | 730,910 | 40,747 | 8,246 | 29,115 | 953,139 | 5,031,781 | 3,523,432 |
|  | 2 | 5,444,477 | 6,678,054 | 369,568 | 731,440 | 41,556 | 8,408 | 29,183 | 953,635 | 5,033,692 | 3,524,490 |
|  | 3 | 5,444,797 | 6,679,263 | 369,809 | 731,839 | 41,853 | 8,507 | 29,208 | 954,053 | 5,034,232 | 3,525,033 |
|  | 4 | 5,446,028 | 6,681,009 | 369,996 | 732,150 | 42,018 | 8,545 | 29,216 | 954,440 | 5,036,036 | 3,526,018 |
|  | 5 | 5,446,824 | 6,681,671 | 370,172 | 732,369 | 42,100 | 8,573 | 29,210 | 954,735 | 5,037,590 | 3,526,347 |
|  | 6 | 5,448,033 | 6,682,585 | 370,397 | 732,528 | 42,142 | 8,586 | 29,244 | 954,966 | 5,039,238 | 3,526,789 |
|  | 7 | 5,449,893 | 6,684,685 | 370,541 | 732,739 | 42,147 | 8,587 | 29,257 | 955,293 | 5,041,259 | 3,527,685 |
|  | 8 | 5,450,591 | 6,685,537 | 370,539 | 732,827 | 42,134 | 8,589 | 29,262 | 955,483 | 5,041,729 | 3,528,204 |
|  | 9 | 5,451,490 | 6,687,421 | 370,555 | 732,906 | 42,118 | 8,588 | 29,250 | 955,686 | 5,042,497 | 3,529,286 |
|  | 10 | 5,452,813 | 6,688,949 | 370,641 | 733,141 | 42,121 | 8,588 | 29,253 | 955,771 | 5,043,540 | 3,530,077 |
| Average | | 5,447,233 | 6,681,864 | 369,982 | 732,138 | 41,690 | 8,489 | 29,206 | 954,567 | 5,037,484 | 3,526,409 |
| Max  heat  index | 0 | 5,449,115 | 6,685,047 | 369,261 | 731,884 | 39,599 | 8,174 | 29,122 | 954,442 | 5,038,601 | 3,528,190 |
|  | 1 | 5,450,218 | 6,684,753 | 369,659 | 732,049 | 40,760 | 8,241 | 29,145 | 954,434 | 5,039,224 | 3,528,193 |
|  | 2 | 5,450,896 | 6,685,663 | 370,021 | 732,347 | 41,524 | 8,387 | 29,204 | 954,728 | 5,040,386 | 3,528,629 |
|  | 3 | 5,450,727 | 6,686,177 | 370,228 | 732,609 | 41,803 | 8,498 | 29,233 | 955,051 | 5,040,289 | 3,528,790 |
|  | 4 | 5,451,561 | 6,687,945 | 370,332 | 732,967 | 42,018 | 8,548 | 29,240 | 955,337 | 5,041,683 | 3,529,780 |
|  | 5 | 5,452,117 | 6,688,543 | 370,532 | 733,124 | 42,108 | 8,578 | 29,229 | 955,532 | 5,042,817 | 3,530,058 |
|  | 6 | 5,452,318 | 6,688,539 | 370,685 | 733,194 | 42,152 | 8,587 | 29,258 | 955,567 | 5,043,162 | 3,530,030 |
|  | 7 | 5,453,513 | 6,689,796 | 370,787 | 733,362 | 42,162 | 8,588 | 29,267 | 955,828 | 5,044,372 | 3,530,597 |
|  | 8 | 5,454,107 | 6,690,714 | 370,814 | 733,441 | 42,154 | 8,589 | 29,270 | 955,992 | 5,044,945 | 3,531,084 |
|  | 9 | 5,454,962 | 6,692,302 | 370,833 | 733,517 | 42,144 | 8,589 | 29,261 | 956,165 | 5,045,736 | 3,532,003 |
|  | 10 | 5,456,400 | 6,694,044 | 370,881 | 733,708 | 42,140 | 8,588 | 29,257 | 956,281 | 5,046,746 | 3,532,906 |
| Average | | 5,452,358 | 6,688,502 | 370,367 | 732,928 | 41,688 | 8,488 | 29,226 | 955,396 | 5,042,542 | 3,530,024 |

|  | **1** | **2** | **3** | **4** | **5** | **6** | **7** | **8** | **9** | **10** | **11** | **12** | **13** | **14** | **15** | **16** | **17** | **18** | **19** | **20** | **21** | **22** | **23** | **24** | **25** | **26** | **27** | **28** | **29** | **30** |
| --- | --- | --- | --- | --- | --- | --- | --- | --- | --- | --- | --- | --- | --- | --- | --- | --- | --- | --- | --- | --- | --- | --- | --- | --- | --- | --- | --- | --- | --- | --- |
| **1** | 1 | -0.76 | 0.94 | 0.09 | 0.23 | 0.51 | -0.41 | -0.44 | -0.25 | -0.55 | 0.11 | -0.22 | 0.13 | 0.16 | 0.07 | 0.32 | 0.02 | 0.09 | 0.18 | -0.32 | 0.12 | -0.38 | -0.43 | -0.32 | -0.26 | -0.21 | 0.26 | -0.08 | -0.03 | -0.17 |
| **2** |  | 1 | -0.67 | -0.06 | -0.03 | -0.42 | 0.29 | 0.49 | 0.39 | 0.45 | -0.03 | 0.4 | 0.08 | 0.07 | -0.17 | -0.35 | -0.1 | -0.11 | -0.25 | 0.08 | -0.18 | 0.43 | 0.37 | 0.33 | 0.43 | 0.4 | -0.19 | 0.2 | 0.21 | 0.1 |
| **3** |  |  | 1 | 0.12 | 0.33 | 0.48 | -0.42 | -0.31 | -0.11 | -0.55 | 0.18 | -0.08 | 0.1 | 0.05 | 0.03 | 0.3 | -0.05 | 0.11 | 0.14 | -0.31 | 0.11 | -0.39 | -0.33 | -0.28 | -0.2 | -0.06 | 0.27 | -0.11 | -0.01 | -0.27 |
| **4** |  |  |  | 1 | 0.07 | 0.06 | 0.03 | -0.3 | -0.25 | -0.23 | 0.13 | -0.09 | -0.05 | 0.16 | 0.07 | -0.22 | -0.31 | -0.3 | -0.28 | 0.11 | -0.18 | 0.25 | 0.1 | 0.14 | 0.11 | -0.24 | -0.12 | 0.21 | -0.07 | 0.24 |
| **5** |  |  |  |  | 1 | 0.12 | -0.19 | 0.19 | 0.11 | 0.45 | -0.22 | -0.38 | -0.44 | -0.05 | -0.61 | 0.61 | 0.39 | 0.6 | 0.42 | -0.45 | 0.47 | -0.49 | -0.5 | -0.2 | -0.62 | 0.01 | 0.16 | -0.61 | 0.03 | -0.72 |
| **6** |  |  |  |  |  | 1 | -0.95 | -0.35 | -0.13 | -0.2 | -0.15 | -0.08 | 0.31 | 0.23 | -0.1 | 0.32 | 0.2 | 0.17 | 0.24 | -0.54 | 0.19 | -0.45 | -0.46 | -0.63 | -0.23 | -0.12 | 0.31 | 0 | -0.62 | -0.13 |
| **7** |  |  |  |  |  |  | 1 | 0.06 | -0.05 | 0.05 | 0.16 | -0.02 | -0.23 | -0.14 | 0.19 | -0.36 | -0.26 | -0.29 | -0.29 | 0.61 | -0.24 | 0.5 | 0.48 | 0.63 | 0.23 | -0.04 | -0.33 | 0.1 | 0.58 | 0.27 |
| **8** |  |  |  |  |  |  |  | 1 | 0.61 | 0.51 | 0 | 0.35 | -0.32 | -0.3 | -0.27 | 0.05 | 0.16 | 0.36 | 0.1 | -0.12 | 0.12 | -0.06 | 0.05 | 0.14 | 0.06 | 0.53 | -0.01 | -0.29 | 0.27 | -0.41 |
| **9** |  |  |  |  |  |  |  |  | 1 | 0.32 | 0.06 | 0.47 | -0.05 | -0.35 | -0.09 | 0.02 | 0.05 | 0.38 | 0.27 | -0.15 | 0.17 | 0.14 | 0.13 | 0.27 | 0.3 | 0.95 | -0.16 | -0.21 | 0.2 | -0.35 |
| **10** |  |  |  |  |  |  |  |  |  | 1 | -0.38 | -0.17 | -0.44 | -0.29 | -0.52 | 0.5 | 0.63 | 0.67 | 0.51 | -0.32 | 0.51 | -0.29 | -0.32 | -0.03 | -0.45 | 0.18 | -0.12 | -0.58 | -0.12 | -0.54 |
| **11** |  |  |  |  |  |  |  |  |  |  | 1 | 0.28 | 0.24 | 0.11 | 0.02 | -0.45 | -0.47 | -0.35 | -0.51 | 0.35 | -0.43 | 0.32 | 0.27 | 0.09 | 0.41 | 0.06 | -0.01 | 0.45 | 0.3 | 0.31 |
| **12** |  |  |  |  |  |  |  |  |  |  |  | 1 | 0.36 | -0.19 | 0.07 | -0.43 | -0.37 | -0.25 | -0.28 | 0.19 | -0.31 | 0.4 | 0.6 | 0.24 | 0.75 | 0.62 | -0.16 | 0.43 | 0.23 | 0.26 |
| **13** |  |  |  |  |  |  |  |  |  |  |  |  | 1 | 0.35 | 0.16 | -0.38 | -0.38 | -0.41 | -0.33 | 0.11 | -0.35 | 0.32 | 0.29 | -0.07 | 0.57 | 0.07 | 0.03 | 0.52 | 0.01 | 0.5 |
| **14** |  |  |  |  |  |  |  |  |  |  |  |  |  | 1 | 0 | -0.31 | -0.28 | -0.37 | -0.36 | 0.03 | -0.39 | 0.15 | -0.05 | -0.09 | 0.13 | -0.3 | 0.14 | 0.47 | 0.01 | 0.38 |
| **15** |  |  |  |  |  |  |  |  |  |  |  |  |  |  | 1 | -0.37 | -0.34 | -0.39 | -0.15 | 0.26 | -0.28 | 0.29 | 0.35 | 0.21 | 0.32 | -0.02 | -0.04 | 0.29 | 0.04 | 0.41 |
| **16** |  |  |  |  |  |  |  |  |  |  |  |  |  |  |  | 1 | 0.82 | 0.83 | 0.86 | -0.63 | 0.91 | -0.81 | -0.75 | -0.4 | -0.8 | -0.05 | 0.18 | -0.76 | -0.33 | -0.75 |
| **17** |  |  |  |  |  |  |  |  |  |  |  |  |  |  |  |  | 1 | 0.78 | 0.74 | -0.7 | 0.76 | -0.74 | -0.77 | -0.49 | -0.72 | -0.04 | 0.31 | -0.66 | -0.46 | -0.61 |
| **18** |  |  |  |  |  |  |  |  |  |  |  |  |  |  |  |  |  | 1 | 0.85 | -0.65 | 0.75 | -0.7 | -0.66 | -0.31 | -0.65 | 0.25 | 0.14 | -0.8 | -0.21 | -0.83 |
| **19** |  |  |  |  |  |  |  |  |  |  |  |  |  |  |  |  |  |  | 1 | -0.54 | 0.82 | -0.62 | -0.58 | -0.22 | -0.62 | 0.18 | 0.09 | -0.69 | -0.27 | -0.65 |
| **20** |  |  |  |  |  |  |  |  |  |  |  |  |  |  |  |  |  |  |  | 1 | -0.49 | 0.71 | 0.79 | 0.61 | 0.49 | -0.09 | -0.39 | 0.49 | 0.49 | 0.63 |
| **21** |  |  |  |  |  |  |  |  |  |  |  |  |  |  |  |  |  |  |  |  | 1 | -0.6 | -0.57 | -0.23 | -0.59 | 0.13 | 0.09 | -0.67 | -0.29 | -0.63 |
| **22** |  |  |  |  |  |  |  |  |  |  |  |  |  |  |  |  |  |  |  |  |  | 1 | 0.8 | 0.64 | 0.77 | 0.21 | -0.38 | 0.57 | 0.45 | 0.64 |
| **23** |  |  |  |  |  |  |  |  |  |  |  |  |  |  |  |  |  |  |  |  |  |  | 1 | 0.57 | 0.77 | 0.26 | -0.36 | 0.56 | 0.46 | 0.59 |
| **24** |  |  |  |  |  |  |  |  |  |  |  |  |  |  |  |  |  |  |  |  |  |  |  | 1 | 0.46 | 0.33 | -0.65 | 0.16 | 0.66 | 0.22 |
| **25** |  |  |  |  |  |  |  |  |  |  |  |  |  |  |  |  |  |  |  |  |  |  |  |  | 1 | 0.45 | -0.3 | 0.7 | 0.36 | 0.63 |
| **26** |  |  |  |  |  |  |  |  |  |  |  |  |  |  |  |  |  |  |  |  |  |  |  |  |  | 1 | -0.22 | -0.09 | 0.24 | -0.25 |
| **27** |  |  |  |  |  |  |  |  |  |  |  |  |  |  |  |  |  |  |  |  |  |  |  |  |  |  | 1 | -0.03 | -0.39 | -0.06 |
| **28** |  |  |  |  |  |  |  | 24 |  |  |  |  |  |  |  |  |  |  |  |  |  |  |  |  |  |  |  | 1 | 0.13 | 0.86 |
| **29** |  |  |  |  |  |  |  |  |  |  |  |  |  |  |  |  |  |  |  |  |  |  |  |  |  |  |  |  | 1 | 0.09 |
| **30** |  |  |  |  |  |  |  |  |  |  |  |  |  |  |  |  |  |  |  |  |  |  |  |  |  |  |  |  |  | 1 |

**Supplemental Table 2**. Pearson correlation coefficients between independent variables. We highlighted values > 0.8. We also shaded removed variables in the analysis. Because of the long variable names, we replaced them with numbers. Variable names and numbers are followed 1: Median age; 2: Under 5 years; 3: Over 65 years; 4: Over 65 years in nursing facilities; 5: Female; 6: Anglo American; 7: African American; 8: American Indian, Asian, Native Hawaiian, or other races; 9: Hispanic; 10: Labor force; 11: Unemployment rate; 12: Farming, fishing, mining, and forestry; 13: Construction and extraction; 14: Installation, maintenance, and repair; 15: Services; 16: Average income earned per person (Per capita income); 17: Median household income; 18: Median gross rent; 19: Median house value; 20: Households earning $10,000 or less; 21: Households earning $200,000 or more; 22: Households receiving food stamps/SNAP; 23: Population 16 years and older below poverty level; 24: Housing units with no automobile; 25: Population 25 years or older with less than a high school diploma; 26: Population 5 years and older speaking English as a second language with limited English proficiency (those who speak English not very well or not at all); 27: Median year structure built; 28: Housing units that are mobile homes; 29: Children under 18 years living in one-parent families; 30: Population living in rural block groups.

**Supplemental Table 3**. Descriptive summary of socioeconomic variables by county

| County | Median age | Under 5 years | Over 65 years | Over 65 years in nursing facilities | Female | Non-Hispanic White | Non-Hispanic Black | Non-Hispanic other races | Hispanic | Labor force |
| --- | --- | --- | --- | --- | --- | --- | --- | --- | --- | --- |
| Alachua | 30.1 | 5.3 | 10.8 | 0.3 | 51.6 | 70.4 | 20.1 | 9.5 | 8.4 | 59.5 |
| Baker | 35.9 | 6.9 | 10.8 | 0.7 | 47.9 | 84.0 | 14.0 | 2.0 | 2.1 | 54.1 |
| Bay | 39.3 | 6.3 | 14.7 | 0.5 | 50.5 | 82.5 | 10.5 | 7.0 | 4.8 | 63.4 |
| Bradford | 38.7 | 5.9 | 15.1 | 0.7 | 43.2 | 77.0 | 20.6 | 2.4 | 3.6 | 48.4 |
| Brevard | 45.5 | 4.9 | 20.6 | 0.4 | 51.0 | 83.7 | 10.0 | 6.3 | 8.2 | 57.3 |
| Broward | 39.7 | 5.9 | 14.3 | 0.2 | 51.5 | 63.8 | 26.7 | 9.5 | 25.3 | 67.2 |
| Calhoun | 40.1 | 5.5 | 15.8 | 1.2 | 46.6 | 80.6 | 15.3 | 4.1 | 5.2 | 41.1 |
| Charlotte | 55.9 | 3.4 | 34.5 | 0.7 | 51.4 | 90.8 | 5.6 | 3.6 | 5.9 | 44.1 |
| Citrus | 54.2 | 3.8 | 32.1 | 0.7 | 51.6 | 93.4 | 2.9 | 3.7 | 4.7 | 42.8 |
| Clay | 38.2 | 6.1 | 11.9 | 0.3 | 51.0 | 82.9 | 10.0 | 7.1 | 7.8 | 65.2 |
| Collier | 47.1 | 5.3 | 26.7 | 0.3 | 50.6 | 87.3 | 6.6 | 6.1 | 25.8 | 53.9 |
| Columbia | 40.0 | 6.1 | 15.5 | 0.8 | 48.3 | 77.7 | 17.6 | 4.7 | 4.9 | 55.8 |
| DeSoto | 38.5 | 6.4 | 18.1 | 0.1 | 43.5 | 80.7 | 13.0 | 6.2 | 29.9 | 48.9 |
| Dixie | 44.9 | 6.6 | 19.4 | 0.3 | 47.8 | 87.4 | 9.4 | 3.2 | 2.1 | 42.9 |
| Duval | 35.7 | 6.9 | 11.2 | 0.3 | 51.5 | 61.8 | 29.4 | 8.8 | 7.6 | 66.6 |
| Escambia | 37.5 | 6.3 | 14.5 | 0.4 | 50.5 | 69.4 | 22.3 | 8.3 | 4.8 | 62.0 |
| Flagler | 47.7 | 4.9 | 24.7 | 0.2 | 52.0 | 80.8 | 11.5 | 7.7 | 8.8 | 48.6 |
| Franklin | 42.3 | 4.7 | 17.7 | 0.2 | 42.1 | 82.5 | 14.9 | 2.5 | 4.5 | 48.3 |
| Gadsden | 38.9 | 6.8 | 13.5 | 0.2 | 50.6 | 37.0 | 55.5 | 7.4 | 9.7 | 53.7 |
| Gilchrist | 39.7 | 5.0 | 17.2 | 1.1 | 47.5 | 91.9 | 5.5 | 2.6 | 4.8 | 49.6 |
| Glades | 43.4 | 5.8 | 22.1 | 0.0 | 44.1 | 78.6 | 11.7 | 9.7 | 20.9 | 41.9 |
| Gulf | 43.8 | 3.8 | 17.1 | 0.6 | 42.9 | 77.8 | 18.5 | 3.6 | 4.3 | 46.6 |
| Hamilton | 38.4 | 5.4 | 13.3 | 0.4 | 40.6 | 61.1 | 34.7 | 4.2 | 8.8 | 43.3 |
| Hardee | 33.1 | 8.0 | 13.4 | 0.4 | 45.3 | 82.4 | 8.2 | 9.4 | 42.6 | 54.1 |
| Hendry | 32.6 | 8.0 | 11.8 | 0.4 | 46.2 | 66.5 | 13.4 | 20.1 | 49.0 | 59.2 |
| Hernando | 47.8 | 4.8 | 25.9 | 0.2 | 52.2 | 90.2 | 5.3 | 4.5 | 10.4 | 47.8 |
| Highlands | 51.4 | 5.1 | 32.2 | 0.7 | 51.2 | 84.9 | 9.3 | 5.8 | 17.4 | 44.3 |
| Hillsborough | 36.1 | 6.5 | 11.9 | 0.2 | 51.2 | 73.3 | 16.7 | 9.9 | 24.9 | 66.1 |
| Holmes | 42.1 | 5.4 | 16.9 | 0.6 | 46.6 | 90.7 | 5.4 | 3.9 | 2.4 | 44.1 |
| Indian River | 48.9 | 4.7 | 27.5 | 0.2 | 51.7 | 87.3 | 8.9 | 3.8 | 11.2 | 52.5 |
| Jackson | 40.4 | 5.2 | 15.9 | 0.8 | 45.1 | 69.0 | 26.2 | 4.9 | 4.4 | 45.3 |
| Jefferson | 43.6 | 5.5 | 16.8 | 0.8 | 48.1 | 60.2 | 36.9 | 2.9 | 3.7 | 50.1 |
| Lafayette | 33.8 | 6.4 | 12.7 | 0.5 | 43.3 | 80.7 | 15.4 | 3.9 | 10.5 | 53.9 |
| Lake | 45.7 | 5.5 | 24.4 | 0.4 | 51.5 | 83.8 | 9.6 | 6.6 | 12.2 | 54.2 |
| Lee | 45.5 | 5.3 | 23.6 | 0.3 | 50.9 | 84.3 | 8.1 | 7.6 | 18.4 | 54.5 |
| Leon | 29.6 | 5.4 | 9.5 | 0.3 | 52.5 | 63.1 | 30.2 | 6.7 | 5.7 | 66.5 |
| Levy | 45.2 | 5.5 | 19.5 | 0.2 | 50.6 | 87.0 | 9.4 | 3.7 | 7.4 | 49.7 |
| Liberty | 38.2 | 4.6 | 10.5 | 0.0 | 37.6 | 75.9 | 15.9 | 8.2 | 8.3 | 49.3 |
| Madison | 40.2 | 6.4 | 16.2 | 0.8 | 47.4 | 56.6 | 38.9 | 4.5 | 4.7 | 48.4 |
| Manatee | 45.6 | 5.6 | 23.5 | 0.3 | 51.6 | 83.6 | 8.6 | 7.7 | 14.8 | 55.7 |
| Marion | 47.4 | 5.2 | 25.8 | 0.3 | 52.0 | 81.6 | 12.4 | 6.0 | 10.9 | 49.0 |
| Martin | 49.8 | 4.3 | 27.5 | 0.4 | 50.6 | 87.6 | 5.7 | 6.8 | 12.1 | 53.4 |
| Miami-Dade | 38.2 | 6.0 | 14.2 | 0.2 | 51.5 | 74.9 | 19.0 | 6.1 | 64.6 | 62.6 |
| Monroe | 46.4 | 4.5 | 17.4 | 0.1 | 46.6 | 90.3 | 6.5 | 3.2 | 20.7 | 63.4 |
| Nassau | 43.1 | 5.3 | 16.5 | 0.3 | 50.9 | 90.2 | 6.8 | 3.1 | 3.3 | 58.8 |
| Okaloosa | 37.7 | 6.6 | 13.8 | 0.3 | 49.7 | 81.1 | 8.9 | 10.0 | 7.1 | 66.2 |
| Okeechobee | 39.0 | 6.6 | 16.9 | 0.4 | 47.2 | 85.1 | 8.5 | 6.4 | 24.0 | 50.9 |
| Orange | 33.7 | 6.5 | 9.7 | 0.3 | 50.8 | 65.3 | 20.7 | 14.1 | 27.1 | 68.8 |
| Osceola | 35.4 | 6.7 | 11.2 | 0.3 | 50.9 | 75.2 | 11.4 | 13.4 | 45.8 | 64.8 |
| Palm Beach | 43.5 | 5.3 | 21.7 | 0.4 | 51.6 | 76.2 | 17.3 | 6.5 | 19.1 | 60.4 |
| Pasco | 43.8 | 5.5 | 20.9 | 0.4 | 51.4 | 89.6 | 4.4 | 6.0 | 11.8 | 54.2 |
| Pinellas | 46.3 | 4.6 | 21.4 | 0.7 | 52.0 | 83.5 | 10.3 | 6.2 | 8.0 | 59.3 |
| Polk | 39.8 | 6.4 | 18.1 | 0.3 | 51.0 | 78.7 | 14.7 | 6.5 | 17.7 | 56.8 |
| Putnam | 43.2 | 6.3 | 19.1 | 0.4 | 50.3 | 77.5 | 16.5 | 6.1 | 9.1 | 48.9 |
| St. Johns | 42.2 | 5.2 | 15.9 | 0.3 | 51.4 | 89.6 | 5.3 | 5.1 | 5.3 | 62.7 |
| St. Lucie | 42.5 | 5.9 | 20.2 | 0.2 | 51.1 | 73.0 | 18.8 | 8.2 | 16.6 | 56.7 |
| Santa Rosa | 39.2 | 5.9 | 12.9 | 0.2 | 49.5 | 87.5 | 5.2 | 7.3 | 4.4 | 61.8 |
| Sarasota | 52.6 | 3.9 | 31.3 | 0.6 | 52.3 | 91.3 | 4.6 | 4.1 | 7.9 | 51.0 |
| Seminole | 38.3 | 5.4 | 12.2 | 0.3 | 51.5 | 80.7 | 10.9 | 8.5 | 17.3 | 66.8 |
| Sumter | 63.0 | 2.4 | 44.5 | 0.3 | 48.1 | 87.5 | 9.8 | 2.8 | 6.0 | 26.2 |
| Suwannee | 42.2 | 6.2 | 18.4 | 0.8 | 49.2 | 84.0 | 11.6 | 4.4 | 8.7 | 51.8 |
| Taylor | 41.2 | 6.0 | 15.9 | 0.3 | 44.6 | 75.7 | 21.4 | 2.9 | 3.5 | 48.9 |
| Union | 39.8 | 5.0 | 10.2 | 0.0 | 35.6 | 74.3 | 20.8 | 5.0 | 4.9 | 39.2 |
| Volusia | 45.3 | 4.9 | 21.3 | 0.5 | 51.2 | 82.2 | 10.4 | 7.4 | 11.3 | 53.2 |
| Wakulla | 39.5 | 5.7 | 10.9 | 0.0 | 45.3 | 82.7 | 13.9 | 3.3 | 3.3 | 58.8 |
| Walton | 43.0 | 5.8 | 16.4 | 0.4 | 48.8 | 87.5 | 5.5 | 7.0 | 5.3 | 57.0 |
| Washington | 40.8 | 5.5 | 15.6 | 0.0 | 46.2 | 79.9 | 14.4 | 5.7 | 3.1 | 47.7 |
| State average | 42.0 | 5.6 | 18.3 | 0.4 | 48.7 | 79.3 | 14.5 | 6.2 | 12.5 | 53.7 |

**Supplemental Table 3.** (continued)

| County | Unemployment rate | Farming, fishing, mining, and forestry | Construction and extraction | Installation, maintenance, and repair | Services | Average income per person | Median household income | Median gross rent | Median house value | Earning  < $10,000 |
| --- | --- | --- | --- | --- | --- | --- | --- | --- | --- | --- |
| Alachua | 8.6 | 0.4 | 2.9 | 2.4 | 19.4 | 24,741 | 40,644 | 855 | 189,600 | 13.7 |
| Baker | 11.4 | 0.6 | 10.7 | 4.7 | 21.7 | 19,593 | 47,276 | 703 | 137,900 | 6.7 |
| Bay | 9.9 | 0.5 | 7.6 | 4.6 | 19.4 | 25,033 | 47,770 | 889 | 175,500 | 6.0 |
| Bradford | 15.7 | 0.4 | 9.0 | 4.8 | 26.2 | 16,997 | 41,126 | 673 | 120,500 | 10.2 |
| Brevard | 12.4 | 0.2 | 5.8 | 3.9 | 18.0 | 27,606 | 49,523 | 897 | 186,900 | 6.0 |
| Broward | 11.9 | 0.1 | 5.5 | 3.5 | 18.5 | 28,631 | 51,694 | 1,133 | 247,500 | 6.7 |
| Calhoun | 10.2 | 1.9 | 6.5 | 4.7 | 24.4 | 15,091 | 31,699 | 530 | 89,000 | 10.4 |
| Charlotte | 13.7 | 0.4 | 7.6 | 3.9 | 19.0 | 26,938 | 45,037 | 927 | 184,900 | 6.3 |
| Citrus | 15.8 | 0.4 | 6.9 | 5.1 | 23.3 | 22,551 | 37,933 | 749 | 141,800 | 7.7 |
| Clay | 11.3 | 0.3 | 5.7 | 5.5 | 15.4 | 26,872 | 61,185 | 971 | 189,700 | 4.1 |
| Collier | 10.1 | 2.2 | 9.7 | 3.0 | 23.0 | 37,046 | 58,106 | 1,054 | 357,400 | 5.2 |
| Columbia | 18.8 | 0.2 | 7.5 | 6.4 | 17.5 | 19,366 | 38,214 | 669 | 134,300 | 8.4 |
| DeSoto | 12.6 | 17.6 | 10.9 | 3.0 | 21.4 | 15,989 | 35,979 | 715 | 114,100 | 9.0 |
| Dixie | 9.0 | 1.6 | 14.7 | 8.3 | 19.3 | 17,066 | 32,312 | 526 | 98,200 | 6.7 |
| Duval | 11.7 | 0.1 | 5.7 | 3.8 | 17.0 | 25,854 | 49,463 | 880 | 175,900 | 8.0 |
| Escambia | 12.0 | 0.2 | 6.3 | 4.1 | 20.1 | 23,474 | 43,573 | 816 | 148,600 | 8.7 |
| Flagler | 12.4 | 0.0 | 5.2 | 3.2 | 19.3 | 24,939 | 48,090 | 1,032 | 219,100 | 5.8 |
| Franklin | 12.0 | 8.0 | 13.4 | 1.7 | 27.3 | 21,005 | 36,490 | 665 | 177,000 | 12.2 |
| Gadsden | 14.1 | 2.0 | 7.2 | 5.4 | 22.4 | 16,843 | 35,728 | 684 | 104,500 | 15.8 |
| Gilchrist | 16.5 | 2.7 | 9.3 | 6.4 | 21.1 | 18,309 | 37,039 | 738 | 112,700 | 10.7 |
| Glades | 15.8 | 14.5 | 9.2 | 5.3 | 23.9 | 17,872 | 39,429 | 744 | 106,900 | 9.9 |
| Gulf | 11.9 | 1.6 | 10.5 | 4.2 | 23.9 | 17,968 | 39,178 | 739 | 131,900 | 10.2 |
| Hamilton | 16.3 | 3.8 | 7.4 | 3.2 | 23.8 | 15,794 | 37,613 | 528 | 75,700 | 15.4 |
| Hardee | 13.9 | 19.4 | 12.8 | 3.5 | 15.7 | 14,668 | 37,466 | 775 | 111,000 | 6.3 |
| Hendry | 15.4 | 23.2 | 6.5 | 2.5 | 19.4 | 14,734 | 37,298 | 764 | 117,300 | 9.9 |
| Hernando | 17.2 | 0.5 | 8.1 | 3.8 | 19.0 | 22,775 | 42,011 | 864 | 156,400 | 7.2 |
| Highlands | 15.2 | 5.5 | 8.2 | 4.2 | 20.8 | 19,579 | 34,946 | 715 | 122,000 | 8.3 |
| Hillsborough | 11.0 | 0.9 | 5.2 | 3.3 | 16.7 | 27,062 | 49,536 | 906 | 198,900 | 7.2 |
| Holmes | 11.1 | 2.1 | 10.4 | 5.1 | 21.4 | 15,285 | 32,247 | 611 | 87,100 | 12.2 |
| Indian River | 14.7 | 1.9 | 9.3 | 3.4 | 21.1 | 31,918 | 47,341 | 899 | 198,200 | 5.4 |
| Jackson | 12.9 | 0.8 | 6.3 | 3.9 | 27.3 | 17,177 | 38,257 | 554 | 97,700 | 11.1 |
| Jefferson | 14.9 | 3.5 | 5.2 | 3.0 | 22.8 | 19,647 | 41,359 | 693 | 127,600 | 10.5 |
| Lafayette | 11.2 | 4.9 | 7.7 | 3.1 | 27.4 | 18,069 | 46,445 | 617 | 162,200 | 7.2 |
| Lake | 12.0 | 0.8 | 7.0 | 3.9 | 19.7 | 25,323 | 46,477 | 904 | 178,400 | 5.3 |
| Lee | 13.5 | 0.4 | 8.8 | 4.3 | 20.1 | 29,445 | 50,014 | 962 | 210,600 | 5.8 |
| Leon | 11.2 | 0.2 | 4.1 | 2.4 | 17.1 | 25,803 | 44,490 | 879 | 196,700 | 13.0 |
| Levy | 14.8 | 4.0 | 10.7 | 5.5 | 18.5 | 18,703 | 35,737 | 618 | 112,100 | 11.3 |
| Liberty | 20.6 | 3.6 | 11.7 | 3.5 | 17.0 | 17,003 | 40,777 | 685 | 90,700 | 13.4 |
| Madison | 14.2 | 4.0 | 8.5 | 3.0 | 19.9 | 16,346 | 37,459 | 648 | 96,400 | 10.6 |
| Manatee | 11.3 | 1.2 | 6.7 | 3.4 | 18.0 | 28,072 | 47,812 | 930 | 214,000 | 5.8 |
| Marion | 13.8 | 1.5 | 6.8 | 4.4 | 20.4 | 22,384 | 40,339 | 829 | 150,700 | 7.7 |
| Martin | 12.8 | 1.3 | 7.0 | 3.4 | 19.7 | 35,772 | 53,210 | 1,001 | 254,900 | 5.3 |
| Miami-Dade | 11.8 | 0.5 | 6.9 | 3.2 | 19.9 | 22,957 | 43,605 | 1,004 | 269,600 | 10.1 |
| Monroe | 7.5 | 2.3 | 6.5 | 3.8 | 23.9 | 35,516 | 53,821 | 1,257 | 503,900 | 6.2 |
| Nassau | 10.1 | 0.6 | 9.7 | 5.1 | 16.5 | 29,089 | 58,712 | 859 | 213,600 | 5.6 |
| Okaloosa | 8.8 | 0.4 | 6.4 | 4.5 | 20.4 | 28,621 | 54,242 | 962 | 204,400 | 5.6 |
| Okeechobee | 15.9 | 5.2 | 9.1 | 2.9 | 24.7 | 19,664 | 38,339 | 788 | 136,700 | 6.6 |
| Orange | 11.4 | 0.3 | 6.3 | 2.8 | 19.8 | 25,490 | 50,138 | 995 | 228,600 | 6.4 |
| Osceola | 12.8 | 0.5 | 6.7 | 4.0 | 23.1 | 20,536 | 46,328 | 1,036 | 199,200 | 5.4 |
| Palm Beach | 11.8 | 0.9 | 6.4 | 3.0 | 20.4 | 33,610 | 53,242 | 1,129 | 261,900 | 6.3 |
| Pasco | 12.1 | 0.4 | 6.3 | 4.6 | 17.9 | 24,164 | 44,228 | 865 | 157,400 | 6.1 |
| Pinellas | 10.5 | 0.1 | 4.8 | 3.1 | 16.8 | 28,742 | 45,258 | 904 | 185,700 | 6.8 |
| Polk | 12.6 | 1.5 | 7.4 | 3.9 | 19.2 | 21,881 | 43,946 | 835 | 141,900 | 7.1 |
| Putnam | 14.1 | 3.6 | 9.4 | 4.2 | 18.0 | 18,402 | 34,645 | 587 | 109,300 | 12.7 |
| St. Johns | 7.8 | 0.3 | 4.4 | 2.7 | 16.4 | 36,027 | 62,663 | 1,025 | 294,100 | 4.9 |
| St. Lucie | 15.0 | 1.2 | 8.5 | 4.6 | 20.6 | 23,296 | 45,196 | 1,014 | 177,200 | 6.5 |
| Santa Rosa | 11.0 | 0.5 | 7.7 | 4.4 | 18.6 | 25,384 | 55,129 | 900 | 182,300 | 5.4 |
| Sarasota | 11.4 | 0.1 | 7.4 | 3.5 | 19.8 | 33,045 | 49,388 | 1,004 | 235,100 | 5.5 |
| Seminole | 10.7 | 0.2 | 5.0 | 2.8 | 14.4 | 29,795 | 58,971 | 1,024 | 241,000 | 5.0 |
| Sumter | 13.2 | 1.1 | 8.4 | 4.1 | 23.3 | 24,180 | 43,079 | 688 | 184,000 | 5.8 |
| Suwannee | 11.7 | 3.2 | 8.3 | 5.6 | 19.2 | 18,782 | 36,352 | 650 | 111,600 | 10.3 |
| Taylor | 13.2 | 0.9 | 6.8 | 4.7 | 20.5 | 18,649 | 37,408 | 534 | 87,800 | 11.2 |
| Union | 9.6 | 0.6 | 6.9 | 4.6 | 35.5 | 13,657 | 41,794 | 560 | 115,100 | 8.8 |
| Volusia | 11.2 | 0.8 | 6.8 | 4.2 | 20.3 | 24,768 | 44,400 | 879 | 186,300 | 7.8 |
| Wakulla | 10.5 | 1.1 | 9.2 | 4.3 | 18.6 | 21,892 | 53,301 | 845 | 143,500 | 6.8 |
| Walton | 9.9 | 0.5 | 10.6 | 4.4 | 19.2 | 27,746 | 47,273 | 889 | 199,800 | 7.8 |
| Washington | 13.5 | 1.0 | 8.0 | 5.8 | 21.3 | 18,470 | 36,216 | 616 | 103,800 | 11.6 |
| State average | 12.6 | 2.5 | 7.8 | 4.1 | 20.5 | 23,130 | 44,269 | 818 | 169,796 | 8.2 |

**Supplemental Table 3**. (continued)

| County | Earning > 200,000 | Food stamps/  SNAP | Below poverty level | No automobile | Less than a high school diploma; | English proficiency | Median year structure built | Mobile homes | One-parent families | Rural block groups |
| --- | --- | --- | --- | --- | --- | --- | --- | --- | --- | --- |
| Alachua | 3.5 | 7.6 | 23.6 | 8.4 | 10.3 | 1.6 | 1985 | 8.7 | 31.7 | 21.0 |
| Baker | 2.4 | 9.1 | 17.0 | 3.0 | 21.6 | 0.2 | 1989 | 41.4 | 22.2 | 59.0 |
| Bay | 2.0 | 7.8 | 12.4 | 6.2 | 13.7 | 1.3 | 1986 | 13.4 | 35.0 | 11.6 |
| Bradford | 1.6 | 13.2 | 16.0 | 9.1 | 23.6 | 0.3 | 1981 | 30.1 | 32.7 | 71.5 |
| Brevard | 3.0 | 6.1 | 10.5 | 5.7 | 9.4 | 1.2 | 1985 | 8.7 | 34.2 | 5.0 |
| Broward | 4.4 | 7.6 | 12.3 | 7.5 | 12.9 | 7.2 | 1979 | 2.9 | 36.6 | 0.0 |
| Calhoun | 1.3 | 16.3 | 21.1 | 7.8 | 25.9 | 1.5 | 1984 | 33.1 | 30.9 | 77.5 |
| Charlotte | 2.1 | 5.3 | 10.5 | 4.8 | 11.7 | 1.3 | 1988 | 11.8 | 32.5 | 8.9 |
| Citrus | 1.6 | 7.3 | 14.4 | 5.7 | 15.0 | 0.7 | 1987 | 27.0 | 36.2 | 35.0 |
| Clay | 3.0 | 5.6 | 8.5 | 3.1 | 9.7 | 1.3 | 1990 | 13.3 | 27.4 | 16.2 |
| Collier | 8.1 | 4.2 | 12.2 | 5.4 | 15.2 | 10.2 | 1992 | 5.7 | 33.3 | 7.2 |
| Columbia | 1.0 | 13.0 | 15.6 | 6.0 | 18.0 | 0.9 | 1988 | 38.6 | 35.8 | 64.8 |
| DeSoto | 1.1 | 12.6 | 26.9 | 7.4 | 32.5 | 14.4 | 1983 | 34.3 | 37.4 | 42.7 |
| Dixie | 0.7 | 12.1 | 15.5 | 7.7 | 27.4 | 1.5 | 1986 | 51.3 | 37.4 | 87.7 |
| Duval | 2.7 | 8.8 | 14.2 | 8.4 | 12.8 | 2.3 | 1982 | 5.4 | 39.2 | 2.5 |
| Escambia | 2.5 | 10.7 | 16.4 | 6.1 | 13.3 | 1.2 | 1980 | 7.7 | 35.9 | 9.9 |
| Flagler | 2.3 | 4.9 | 11.9 | 3.9 | 9.8 | 2.1 | 1998 | 4.9 | 34.1 | 10.5 |
| Franklin | 1.3 | 14.2 | 25.6 | 6.2 | 21.7 | 1.3 | 1982 | 19.3 | 35.7 | 75.8 |
| Gadsden | 0.7 | 17.8 | 27.6 | 11.2 | 24.3 | 2.7 | 1983 | 28.5 | 58.3 | 68.5 |
| Gilchrist | 1.3 | 11.7 | 20.1 | 5.2 | 18.9 | 0.9 | 1991 | 51.7 | 24.8 | 90.1 |
| Glades | 0.8 | 6.7 | 19.6 | 5.8 | 28.7 | 9.6 | 1986 | 57.2 | 39.2 | 80.5 |
| Gulf | 0.8 | 12.3 | 19.5 | 4.9 | 22.3 | 0.6 | 1985 | 21.7 | 31.6 | 79.7 |
| Hamilton | 1.7 | 18.5 | 21.4 | 10.9 | 26.1 | 2.4 | 1983 | 41.9 | 39.3 | 78.9 |
| Hardee | 0.6 | 16.9 | 26.1 | 7.3 | 40.1 | 16.1 | 1981 | 29.6 | 35.0 | 49.2 |
| Hendry | 1.3 | 16.3 | 26.4 | 9.7 | 38.0 | 18.4 | 1986 | 39.0 | 44.0 | 38.5 |
| Hernando | 1.5 | 8.5 | 11.8 | 4.5 | 14.4 | 1.4 | 1988 | 18.2 | 33.4 | 20.2 |
| Highlands | 0.7 | 8.6 | 16.9 | 6.4 | 21.2 | 4.8 | 1985 | 27.5 | 34.0 | 22.0 |
| Hillsborough | 3.8 | 8.5 | 14.2 | 7.1 | 14.2 | 5.3 | 1985 | 8.0 | 37.2 | 3.2 |
| Holmes | 0.1 | 15.3 | 19.9 | 7.0 | 25.9 | 0.5 | 1982 | 32.0 | 32.6 | 85.3 |
| Indian River | 5.1 | 6.1 | 12.6 | 5.4 | 13.7 | 3.9 | 1990 | 8.4 | 28.4 | 4.2 |
| Jackson | 1.2 | 11.0 | 19.7 | 9.5 | 22.6 | 1.3 | 1981 | 27.4 | 43.4 | 81.3 |
| Jefferson | 1.9 | 13.8 | 18.7 | 6.5 | 20.1 | 0.8 | 1988 | 35.7 | 40.0 | 100.0 |
| Lafayette | 0.4 | 9.9 | 18.0 | 6.7 | 18.7 | 1.8 | 1988 | 39.5 | 18.6 | 100.0 |
| Lake | 2.2 | 6.1 | 11.0 | 5.5 | 13.1 | 2.0 | 1992 | 22.6 | 35.5 | 18.1 |
| Lee | 4.1 | 5.8 | 12.0 | 5.1 | 13.3 | 5.8 | 1991 | 10.9 | 37.4 | 6.5 |
| Leon | 2.9 | 8.4 | 22.0 | 6.5 | 9.4 | 0.9 | 1986 | 9.1 | 36.4 | 11.7 |
| Levy | 1.3 | 15.8 | 21.6 | 6.6 | 19.7 | 1.5 | 1988 | 45.7 | 39.6 | 93.3 |
| Liberty | 1.3 | 10.3 | 15.8 | 5.5 | 25.1 | 0.5 | 1984 | 46.1 | 35.3 | 100.0 |
| Madison | 1.2 | 16.8 | 21.0 | 10.6 | 25.2 | 1.8 | 1980 | 35.3 | 44.5 | 79.4 |
| Manatee | 3.4 | 5.8 | 12.8 | 5.3 | 13.1 | 4.3 | 1984 | 17.2 | 35.0 | 4.0 |
| Marion | 1.9 | 8.9 | 15.3 | 5.4 | 15.5 | 2.1 | 1990 | 22.2 | 38.3 | 30.5 |
| Martin | 6.5 | 3.8 | 10.4 | 4.7 | 11.4 | 4.1 | 1985 | 10.3 | 29.1 | 6.7 |
| Miami-Dade | 4.0 | 16.7 | 17.2 | 11.0 | 23.0 | 22.3 | 1976 | 1.5 | 37.6 | 0.4 |
| Monroe | 5.5 | 5.3 | 10.8 | 7.7 | 10.2 | 4.8 | 1979 | 15.0 | 32.5 | 11.8 |
| Nassau | 4.0 | 7.4 | 9.3 | 4.5 | 13.5 | 0.3 | 1989 | 21.4 | 30.3 | 42.2 |
| Okaloosa | 3.6 | 6.8 | 10.6 | 4.8 | 9.3 | 1.9 | 1986 | 6.8 | 28.0 | 10.1 |
| Okeechobee | 2.3 | 12.0 | 19.7 | 4.1 | 28.4 | 7.7 | 1984 | 41.1 | 32.4 | 36.0 |
| Orange | 3.6 | 7.6 | 13.4 | 6.5 | 13.1 | 6.5 | 1988 | 4.4 | 35.6 | 1.9 |
| Osceola | 1.7 | 10.7 | 13.3 | 5.7 | 15.6 | 9.1 | 1995 | 10.4 | 33.9 | 15.0 |
| Palm Beach | 6.2 | 5.6 | 12.2 | 6.6 | 13.2 | 6.4 | 1984 | 2.9 | 33.8 | 0.9 |
| Pasco | 1.9 | 7.9 | 12.3 | 5.9 | 13.9 | 2.1 | 1986 | 20.8 | 32.1 | 8.5 |
| Pinellas | 3.2 | 6.8 | 12.1 | 8.6 | 11.9 | 2.8 | 1975 | 9.5 | 37.0 | 0.3 |
| Polk | 1.9 | 9.4 | 15.2 | 6.5 | 18.1 | 4.9 | 1986 | 24.1 | 37.3 | 14.3 |
| Putnam | 0.9 | 14.4 | 23.3 | 7.9 | 22.0 | 1.6 | 1981 | 41.0 | 46.7 | 58.8 |
| St. Johns | 7.6 | 4.7 | 9.1 | 3.6 | 7.2 | 0.7 | 1994 | 9.1 | 20.2 | 22.6 |
| St. Lucie | 1.9 | 8.1 | 13.7 | 6.0 | 16.6 | 4.2 | 1989 | 9.6 | 35.2 | 3.6 |
| Santa Rosa | 2.6 | 8.5 | 11.3 | 3.6 | 11.6 | 0.9 | 1992 | 12.9 | 25.2 | 21.4 |
| Sarasota | 4.5 | 4.2 | 10.5 | 5.3 | 9.4 | 2.6 | 1983 | 9.2 | 31.8 | 3.6 |
| Seminole | 4.8 | 4.6 | 9.8 | 3.6 | 9.3 | 2.2 | 1985 | 2.7 | 27.5 | 2.9 |
| Sumter | 1.3 | 5.9 | 11.2 | 3.5 | 15.5 | 1.8 | 2000 | 23.0 | 30.9 | 23.2 |
| Suwannee | 1.5 | 15.2 | 17.3 | 6.4 | 20.1 | 3.3 | 1986 | 48.6 | 35.7 | 84.6 |
| Taylor | 1.4 | 13.2 | 19.1 | 6.4 | 21.3 | 0.3 | 1986 | 37.4 | 26.9 | 71.9 |
| Union | 0.6 | 14.4 | 21.3 | 6.9 | 22.9 | 3.0 | 1986 | 35.6 | 34.7 | 78.0 |
| Volusia | 2.4 | 7.2 | 13.8 | 6.4 | 12.7 | 1.9 | 1984 | 9.4 | 38.5 | 8.7 |
| Wakulla | 0.7 | 5.3 | 12.5 | 4.1 | 15.5 | 0.3 | 1992 | 36.4 | 28.6 | 73.1 |
| Walton | 4.3 | 8.2 | 14.6 | 5.6 | 15.7 | 1.4 | 1994 | 15.4 | 31.9 | 51.3 |
| Washington | 1.2 | 9.2 | 19.3 | 6.0 | 21.3 | 0.9 | 1983 | 32.9 | 34.5 | 83.8 |
| State average | 2.5 | 9.7 | 16.0 | 6.3 | 17.8 | 3.6 | 1986 | 22.8 | 34.3 | 38.6 |

**Supplemental Table 4**. MLR/spatial lag model results with all independent variables. (ED: emergency department; HSP hospital admissions; MLR: multiple linear regression) (* ≤ 0.05).

|  | Cardiovascular  disease | | Dehydration | | Heat-related illness | | Acute renal disease | | Respiratory disease | |
| --- | --- | --- | --- | --- | --- | --- | --- | --- | --- | --- |
|  | ED | HSP | ED | HSP | ED | HSP | ED | HSP | ED | HSP |
| Model type | Spatial lag | MLR | MLR | MLR | MLR | - | - | MLR | Spatial lag | Spatial lag |
| Under 5 years | 0.003 | 0.004 | 0.000 | 0.006 | 0.007 | - | - | -0.003 | 0.004 | 0.004 |
| Over 65 years | 0.001 | 0.001 | -0.001 | 0.001 | 0.012* | - | - | 0.000 | 0.001 | 0.002* |
| Over 65 years in nursing facilities | 0.010* | -0.001 | 0.010 | 0.010 | 0.021 | - | - | 0.018* | 0.013* | 0.004 |
| Female | 0.000 | -0.002 | 0.003 | 0.002 | -0.017* | - | - | 0.001 | -0.001 | -0.002* |
| Non-Hispanic Black | 0.000 | 0.000 | 0.000 | 0.000 | 0.001 | - | - | 0.000 | 0.000 | 0.000 |
| Non-Hispanic other races (American Indian, Asian, Native Hawaiian, or other races) | 0.000 | -0.001 | 0.000 | 0.001 | -0.003 | - | - | 0.000 | 0.000 | 0.000 |
| Hispanic | 0.000 | 0.000 | 0.000 | 0.000 | 0.001 | - | - | 0.000 | 0.000 | 0.000 |
| Labor force | 0.000 | 0.000 | -0.001 | 0.000 | 0.010* | - | - | 0.000 | 0.000 | 0.001* |
| Unemployment rate | -0.001 | -0.001 | 0.000 | -0.001 | -0.006 | - | - | -0.002* | 0.000 | -0.001 |
| Farming, fishing, mining, and forestry | 0.000 | 0.000 | 0.005* | -0.002 | -0.002 | - | - | 0.000 | -0.001* | -0.001 |
| Construction and extraction | -0.001 | -0.001 | -0.002 | 0.005* | -0.006 | - | - | 0.003* | 0.000 | 0.000 |
| Installation, maintenance, and repair | -0.003* | -0.002 | 0.009 | -0.011* | -0.001 | - | - | -0.005* | -0.003* | -0.004* |
| Services | 0.000 | -0.001 | 0.004 | 0.000 | -0.006 | - | - | 0.000 | -0.001* | -0.001 |
| Average income earned per person (Per capita income) | 0.000 | 0.000 | 0.002 | -0.001 | -0.007* | - | - | -0.001 | 0.000 | -0.001* |
| Households earning $10,000 or less | 0.000 | 0.001 | 0.000 | 0.000 | 0.007 | - | - | -0.002 | 0.001 | 0.002* |
| Housing units with no automobile | -0.002 | -0.001 | -0.002 | 0.004 | -0.012 | - | - | 0.000 | -0.002 | -0.002 |
| Median year structure built | 0.000 | 0.000 | -0.001 | 0.000 | 0.001 | - | - | 0.000 | 0.000 | 0.000 |
| Children under 18 years living in one-parent families | 0.001* | 0.000 | -0.002 | -0.001 | 0.002 | - | - | 0.000 | 0.000 | 0.000 |


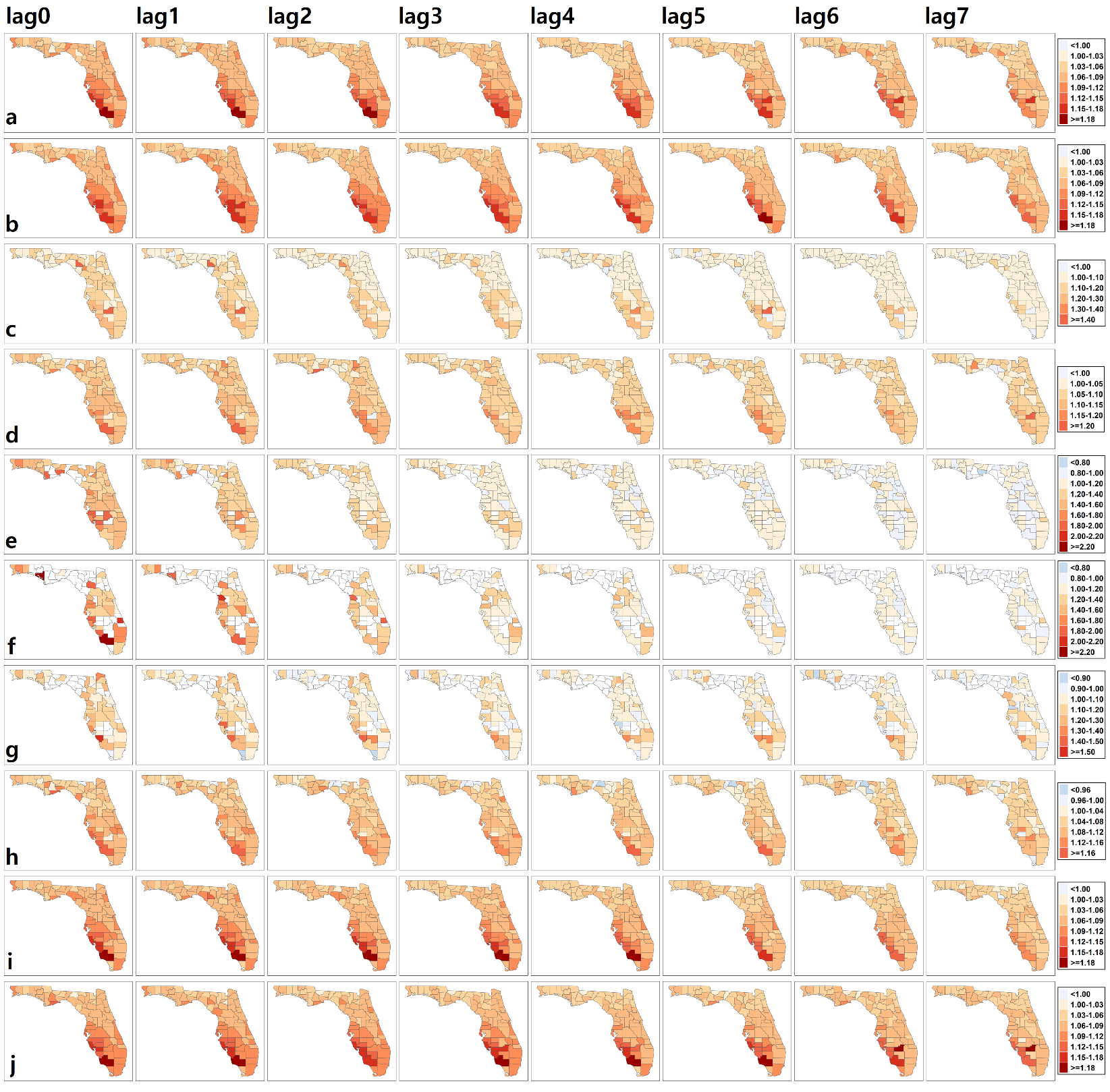


**Supplemental Figure 1.** ORs at the county level. Counties having less than 30 cases were colored in white. a: cardiovascular disease emergency department visits; b: cardiovascular disease hospital admissions; c: dehydration emergency department visits; d: dehydration hospital admissions; e: heat-related illness emergency department visits; f: heat-related illness hospital admissions; g: acute renal disease emergency department visits; h: acute renal disease hospital admissions; i: respiratory disease emergency department visits; j: respiratory disease hospital admissions
